# Transcriptome assemblies for studying sex-biased gene expression in the guppy, *Poecilia reticulata*

**DOI:** 10.1186/1471-2164-15-400

**Published:** 2014-05-26

**Authors:** Eshita Sharma, Axel Künstner, Bonnie A Fraser, Gideon Zipprich, Verena A Kottler, Stefan R Henz, Detlef Weigel, Christine Dreyer

**Affiliations:** Department of Molecular Biology, Max Planck Institute for Developmental Biology, Spemannstrasse 37, 72076 Tübingen, Germany; Bioinformatics and Statistical Genetics Core, Wellcome Trust Centre for Human Genetics, Roosevelt Drive, OX3 7BN Oxford, UK; Division of Theoretical Bioinformatics, German Cancer Research Center, Im Neuenheimer Feld 280, 69120 Heidelberg, Germany

**Keywords:** Guppy, *de novo* transcriptome, Genome-guided transcriptome, Sex-biased genes, Sexual dimorphism, RNA-seq, Coding sequence evolution

## Abstract

**Background:**

Sexually dimorphic phenotypes are generally associated with differential gene expression between the sexes. The study of molecular evolution and genomic location of these differentially expressed, or sex-biased, genes is important for understanding inter-sexual divergence under sex-specific selection pressures. Teleost fish provide a unique opportunity to examine this divergence in the presence of variable sex-determination mechanisms of recent origin. The guppy, *Poecilia reticulata*, displays sexual dimorphism in size, ornaments, and behavior, traits shaped by natural and sexual selection in the wild.

**Results:**

To gain insight into molecular mechanisms underlying the guppy’s sexual dimorphism, we assembled a reference transcriptome combining genome-independent as well as genome-guided assemblies and analyzed sex-biased gene expression between different tissues of adult male and female guppies. We found tissue-associated sex-biased expression of genes related to pigmentation, signal transduction, and spermatogenesis in males; and growth, cell-division, extra-cellular matrix organization, nutrient transport, and folliculogenesis in females. While most sex-biased genes were randomly distributed across linkage groups, we observed accumulation of ovary-biased genes on the sex linkage group, LG12. Both testis-biased and ovary-biased genes showed a significantly higher rate of non-synonymous to synonymous substitutions (*d*_*N*_*/d*_*S*_) compared to unbiased genes. However, in somatic tissues only female-biased genes, including those co-expressed in multiple tissues, showed elevated ratios of non-synonymous substitutions.

**Conclusions:**

Our work identifies a set of annotated gene products that are candidate factors affecting sexual dimorphism in guppies. The differential genomic distribution of gonad-biased genes provides evidence for sex-specific selection pressures acting on the nascent sex chromosomes of the guppy. The elevated rates of evolution of testis-biased and female-biased genes indicate differing evolution under distinct selection pressures on the reproductive versus non-reproductive tissues.

**Electronic supplementary material:**

The online version of this article (doi:10.1186/1471-2164-15-400) contains supplementary material, which is available to authorized users.

## Background

In sexually reproducing species, males and females evolve differently due to differing regimes of natural and sexual selection [1–3]. Nonetheless, the evolution of sexually dimorphic traits within a species is constrained because most of the genome is shared between males and females. Therefore, the development of sex-specific traits is thought to be predominantly accomplished by sex-specific gene expression [4–8]. Quantitative analyses of complementary DNA (cDNA) from male and female tissues of mice (*Mus musculus*) [9], zebrafish (*Danio rerio*) [10], birds (chicken (*Gallus gallus*) [11] and turkey (*Meleagris gallopavo*) [12]), and insects (*Drosophila* species and *Bombyx mori*[13, 14]) have shown that a significant fraction of autosomal genes are differentially expressed between the sexes in their reproductive as well as non-reproductive tissues. This suggests that sex-biased gene expression contributes to sexually dimorphic phenotypes and sex-biased genes may evolve differently under selection pressures acting on the sexual phenotypes [15]. Research on evolutionary properties of sex-biased genes has shown accelerated rates of coding sequence changes in reproduction-related male-biased genes [16, 17]. This is primarily attributed to greater sexual selection on males than females. Elevated nucleotide substitution rates of sex-biased genes expressed in somatic and reproductive tissues may also occur due to relaxed selection on non-pleiotropic tissue-specific genes [18, 19]. Sex-biased genes also show non-random genomic distribution with X- or Z-linkage [20, 21] that can arise due to differential selection on the hemizygous sex chromosome [6, 7].

So far, sex-biased gene expression has mainly been explored in species with well-differentiated sex chromosomes, while species with young or undifferentiated sex-chromosome systems are just beginning to be studied [22–24]. In this regard, teleost fish with their spectacular diversity of sex determination mechanisms and a large repertoire of duplicated genes provide largely unexplored resources to study sexual dimorphism resulting from sex-biased and sex-limited gene expression [25]. Among teleosts, members of the family Poeciliidae are known to have multiple sex determination systems [26, 27] and are characterized by highly variable sexually dimorphic traits including color patterns, body size, genital morphology, and mating behavior [28–30]. The guppy (*Poecilia reticulata*) was one of the first vertebrates where XY sex determination and Y-linked inheritance of sexually selected traits were demonstrated [31]. Sexual dimorphism in guppies is characterized by male-specific color patterns that attract females but are disadvantageous in the presence of predators [32–35]. These male-advantageous traits are believed to have co-evolved with female mate-choice preferences [36, 37]. The guppy also displays sexual size dimorphism. Female guppies grow throughout their life, whereas males slow down their growth during maturation [38]. Male and female guppies also display behavioral differences in the amount of time spent mating, foraging, shoaling, and avoiding predators [32, 39–44].

While the evolutionary ecology of the guppy’s sexual dimorphism has been well studied with respect to heredity and adaptation, the molecular mechanisms governing this dimorphism are largely unidentified. Recently, using a high-density linkage map, quantitative trait loci (QTL) influencing male size, shape, and color traits were mapped to several sex-linked and autosomal loci [45]. Nevertheless, in order to understand the contribution of sex-biased gene regulation to sexually dimorphic phenotypes, a genome-wide comparison of gene expression in sexually dimorphic tissues is required.

Current transcriptome resources of the guppy include a database of Sanger-sequenced expressed sequence tags (EST) and a more recent 454 sequenced transcriptome, that together correspond to roughly 9,000 unique transcripts from embryos and adult guppies originating from several different populations [46, 47].

Here we extend these resources by assembling a reference transcriptome using high depth Illumina sequencing. We used cDNA from multiple tissues from embryos and adults from a single guppy population, thereby minimizing population-specific effects in phenotypic variations and X- and Y-linkage [48, 49]. We then combined the predicted coding sequences from both genome-independent and genome-guided assemblers. The merged reference comprises expressed sequences from embryos and differentiated adult tissues. The transcriptome reference was then used to identify genes with either male- or female-biased expression in three tissues with phenotypic sexual dimorphism in the adult guppy. These included two somatic tissues (brain and tail) and the gonads. Furthermore, by examining sex-biased genes we determined whether i) the expression bias in adult guppy tissues reflects the morphological and physiological differences between the sexes; ii) there is non-random genomic distribution of these genes; and iii) they show signatures of relaxed selection when compared to unbiased genes, as hypothesized for genes subject to sexual selection.

## Results

### Genome-guided and genome-independent transcriptome assemblies

**Figure 1:**
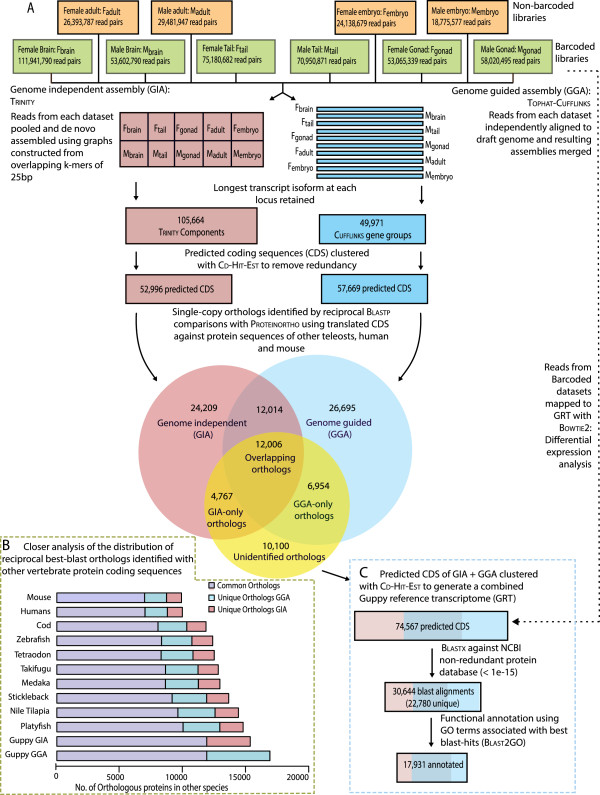
**Assembly of the guppy reference transcriptome. (A)** Flowchart describing read summary, assembly strategy, and assembler comparison. The high quality paired reads from each sequenced dataset, non-barcoded (orange) and barcoded (green), were assembled using genome-independent (Trinity, GIA, red) and genome-guided (Cufflinks, GGA, blue) assemblers. Venn diagram shows the total number of protein sequence orthologs identified between at least two species using translated sequences from the two guppy assemblies (red, blue), and protein sequence databases from eight teleosts, mouse, and human (yellow); **(B)** Inset (dotted yellow, bottom left) shows an alternate view of the ortholog comparisons. Barplots show the number of orthologs identified in two-way reciprocal best blast-hit comparison between platyfish, tilapia, medaka, stickleback, takifugu, tetraodon, zebrafish, cod, human, and mouse proteins. The stacked bars show the number of orthologs common between GGA and GIA (purple), unique to GGA (blue) and unique to GIA (red); **(C)** Inset rectangle (dotted blue, bottom right) summarizes the steps for merging predicted CDS from both assemblies and functional annotation of the guppy reference transcriptome (GRT).

**Figure 2:**
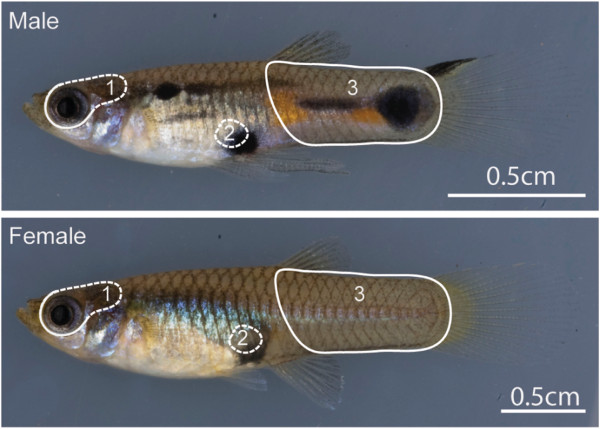
**Phenotypic sexual dimorphism in the guppy.** Males (top) are smaller than females (bottom) and have complex color patterns on the body. The encircled region (white outline) indicates the tissues that were used for preparing the barcoded libraries, 1) brain and eyes; 2) Male testis and female ovary; and 3) tail.

To generate a comprehensive reference transcriptome of the guppy and to investigate gene expression variations between the sexes, we prepared a non-barcoded and a barcoded set of Illumina RNA-seq libraries (Figure 1A). To ensure high complexity cDNA for the reference assembly, the first set of libraries represented cDNA in approximately equal amounts prepared from whole embryos and five different adult tissues from several pooled individuals. For a second set cDNA libraries were prepared from brain, tail, and gonads with individual barcodes for each tissue from six different individuals to allow quantitative comparisons (Additional file 1: Table S1, Figure 2). In total we obtained 521 million quality filtered read pairs (mean read length of 97 bp). The combined dataset of sequenced cDNA was used for *de novo* assembly of the reference transcriptome (Figure 1A).

The genome-independent assembly was assembled with TRINITY, and resulted in 213,088 transcribed sequences, with 105,664 unique components including their isoforms. The genome-guided assembly was assembled using a draft female genome (Künstner *et al*., in preparation) and CUFFLINKS and yielded less than half as many contigs, with 91,126 transcribed sequences comprising 49,971 unique gene groups (Table 1). Exact splice variant prediction requires more elaborate algorithms and was not the focus of our study, therefore we used only the longest isoform for each component (Trinity) or gene group (Cufflinks) for further analysis. We use the term ‘transfrag’ for each individual sequence in the assembly and refer to the longest transcribed isoform as ‘transcripts’ (Table 1).

**Table 1:** **Comparison of transcriptome assembled with genome-guided and genome-independent assemblers**

	**Trinity**: Genome-independent assembly (GIA)	**Cufflinks**: Genome-guided assembly (GGA)
Total length (bp)	416,036,223	301,476,740
Length with longest isoforms per locus (bp)	101,831,430	128,048,246
No. of transfrags	213,088	91,126
No. of transcripts (Unique components/gene groups)	105,664	49,971
Mean length (bp)	1,952	3,308
Longest contig (bp)	65,264	61,058
Overall mapping (%)	73.21	73.64
Concordant and unique mapping (%)	62.98	55.10
Total no. of ORFs	53,537	63,520
No. of complete ORFs	29,309	49,535
Mean length ORF (bp)	766	803
Longest ORF (bp)	63,897	54,732
Total length of assembly with CDSs only (bp)	40,889,623	48,745,723
*Annotations*		
Against guppy (GGA against GIA or vice-versa)	40,973 (24,020)	35,147 (24,020)
*Xiphophorus maculatus*	19,680 (13,399)	19,941 (14,934)
*Oryzias latipes*	17,925 (11,102)	18,197 (12,455)
*Gasterosteus aculeatus*	19,139 (11,758)	19,429 (13,096)
Orthologs in only one assembly	4,767	6,954

We compared a number of metrics to determine which assembler performed better. Open reading frame (ORFs) are those with a minimum length of 50 amino acids. The number of annotations obtained for each assembly are given from best Blastp hits against other protein sequence databases (E-value < 1 × 10^−20^). The number of orthologs (brackets) are given from reciprocal best Blastp hits identified using PROTEIN ORTHO.

### Genome-guided assembly resulted in longer transcripts with more complete open reading frames (ORFs)

The genome-guided and genome-independent assemblies were compared using read-based, length-based, and annotation-based metrics. We compared the i) percent of reads remapped to the transcriptome (completeness); ii) the percent of correctly oriented mapped read pairs (accuracy); iii) total length of assembly and mean length of assembled transcripts (contiguity); iv) number and length of predicted ORFs, and v) number of orthologs identified using reciprocal Blastp against other validated protein sequence databases (Table 1).

Mapping the RNA-seq reads to each assembly we found that the genome-independent assembly incorporated a larger number of correctly oriented read pairs as compared to the genome-guided assembly (Table 1). On the other hand, the genome-guided assembly was more contiguous with longer transcripts, greater number of ORFs, and substantially more full-length ORFs (Table 1).

### Transcriptome annotation and alignment to the genome

By examining the number of single-copy orthologs identified from comparing translated coding sequences (CDS) of the guppy against other teleost, human, and mouse protein sequence databases, we identified a greater number of orthologs in the genome-guided assembly than in the genome-independent assembly (Figure 1A, B, Table 1). The total number of orthologs found between guppy and other species was related to divergence between the species (with the exception of medaka, *Oryzias latipes*, possibly due to the smaller size of the medaka protein database) (Figure 1B). We identified 24,020 reciprocal best-blast hits shared between the genome-guided and genome-independent assemblies (Figure 1A, B, and Table 1). For approximately half (12,006) of these overlapping sequences, orthologous protein sequences were identified in other vertebrates. An additional 11,721 vertebrate orthologs were identified from only one of the two assemblies (Figure 1A, B, and Table 1). In addition to the identified orthologs, 30-40% of the remaining translated CDS aligned (alignment length > 50 amino acids) with significant sequence similarity (E-value < 1 × 10^−20^) to protein coding sequences of the other vertebrates (Table 1).

We merged the CDS predicted from transcripts of genome-guided and genome-independent assemblies to obtain a single comprehensive reference combining advantages from both assembly methods. This final dataset consisted of 74,567 sequences, hereafter referred to as the guppy reference transcriptome (GRT) (Figure 1C). In total, 30,643 (41.1% of the GRT) sequences showed significant alignment (Blastx E-value < 1 × 10^−15^) to 22,780 unique protein sequences in the NCBI non-redundant protein database (NR) [50]. Out of these, 17,931 were annotated with functional categories (Gene Ontology: GO terms) (Figure 1C). A complete list of the best-blast hits and GO annotations is given in Additional file 2: Table S2 and Additional file 3: Table S3. A total of 73,518 sequences could be aligned to assembled scaffolds from the female genome. Of these, 67,882 aligned to scaffolds that were assigned to guppy linkage groups. All the sequences that did not align (1044) were from the genome-independent assembly and of these 693 (66.4%) could be aligned to the genome of the closely related platyfish, *Xiphophorus maculatus* (data not shown).

### Tissue-specific variation in the degree of sexually dimorphic expression

The combined CDS database was used as a reference for quantifying differentially expressed sequences (Figure 1C) in the brain (including eyes), tail (post-anal tissue including skin, skeletal muscle, dorsal cord, bone and cartilage), and gonads of adult guppies (Figure 2). By mapping reads to coding sequences instead of transcripts, we tried to increase the accuracy of read assignment to putative genes but lost the information from reads that represent untranslated regions (UTRs). Therefore, we also performed differential expression analysis after mapping reads to both the genome-guided and genome-independent assemblies and to the full-length transcripts in the merged guppy reference transcriptome. Since the four analyses produced similar results (data not shown), we focus only on results obtained by mapping against the predicted CDS, referred to as genes hereafter. We found the highest number of expressed genes in the brain, followed by gonads and then by tail (Table 2). There was a strong correlation in expression within tissue type for non-reproductive tissue between individuals (Spearman’s correlation ρ > 0.85, p < 1 × 10^−10^), suggesting only a few differences between the sexes. As expected, the greatest sex related difference was observed between the adult ovary and testis where overall expression clustered by sex (Additional file 4: Figure S1). The magnitude of differential expression between sexes varied between the reproductive and non-reproductive tissues, therefore we chose tissue-specific medians as the threshold fold-change required for a gene to be identified as sex-biased (FDR < 0.1, Additional file 5: Figure S2). The complete lists of all median-fold sex-biased genes in individual tissues are available in Additional file 6: Table S4, Additional file 7: Table S5 and Additional file 8: Table S6. Functional categories that were over-represented among sex-biased genes in each tissue and co-expressed sex-biased genes in brain and tail are described in Additional file 9: Table S7, Additional file 10: Table S8, Additional file 11: Table S9 and Additional file 12: Table S10.

**Table 2:** **Differentially expressed genes between males and females in brain, gonad, and tail tissue**

	No. of expressed genes	Fold change	Sex-biased genes (%)	Male-biased genes (%)	Female-biased genes (%)
Brain	27612	> 1.2 fold	3611 (13.08)	1305 (4.73)	2306 (8.35)
		> Median fold	2466 (8.93)	702 (2.54)	1764 (6.39)
Tail	18988	> 1.2 fold	2792 (14.70)	1355 (7.14)	1437 (7.57)
		> Median fold	1460 (7.69)	755 (3.98)	705 (3.71)
Gonad	22873	> 1.2 fold	17740 (77.56)	7989 (34.93)	9751 (42.63)
		> Median fold	10060 (43.98)	4891 (21.38)	5163 (22.57)
*Multiple tissues*					
Brain, tail	18415	> 1.2 fold	767 (4.17)	118 (0.64)	619 (3.36)
		> Median fold	362 (1.97)	44 (0.24)	305 (1.66)
Brain, Gonad	19759	> 1.2 fold	1961 (9.92)	286 (1.45)	1024 (5.18)
		> Median fold	851 (4.31)	84 (0.43)	466 (2.36)
Tail, Gonad	16524	> 1.2 fold	1704 (10.31)	373 (2.26)	526 (3.18)
		> Median fold	447 (2.71)	100 (0.61)	126 (0.76)
Brain, Tail, Gonad	16396	> 1.2 fold	470 (2.87)	43 (0.26)	214 (1.31)
		> Median fold	143 (0.87)	11 (0.07)	59 (0.36)

We report the total number of genes expressed, and those that were sex-biased at two different fold-change cutoffs (1.2 fold and median-fold difference within each tissue) and FDR < 0.1. We also report genes that were sex-biased in multiple tissues with the same direction of change between the sexes.

### Greater number of female-biased genes expressed in the guppy brain

**Figure 3:**
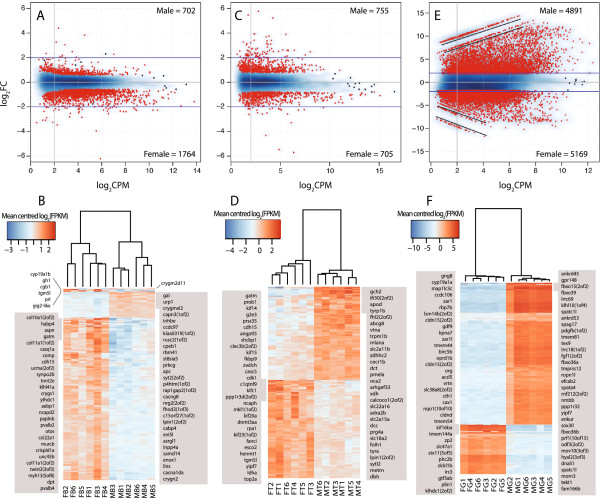
**Quantitative differences in gene expression between sexes.** Male to female expression ratios (log_2_FC, Fold-change: Male/Female) plotted against the average expression intensity (log_2_CPM, Counts per million) in **(A)** brain, **(C)** tail, and **(E)** gonads. Genes with greater than median-fold bias (FDR < 0.1) are shown in red while the others are shown by black dots or smoothened. The blue lines mark a 4-fold difference in expression between the sexes. Genes with sex-limited expression are underlined in black **(E)**. The number of male-biased and female-biased genes in each comparison is mentioned at the top-right and bottom-right respectively in each figure. Heatmaps showing the mean centered log_2_FPKM (Fragments per kilo base per million) for the highest differentially expressed genes (FDR < 0.001) and a 1.5 fold-change in the brain **(B)**, 1.7 fold-change in the tail **(D)**, and 32 fold-change in the gonad **(F)**. The top 30 genes that show sex-biased expression in each tissue are listed and ranked by fold-change in grey text boxes at the left (female-biased genes) and at the right (male-biased genes).

We observed more genes with female-biased expression than with male-biased expression in the brain (Table 2, Figure 3A). Gene ontology (GO) terms enriched among male-biased genes in the brain were related to signal transduction, regulation of transmembrane receptors, and cellular response (Table 3A, Additional file 9: Table S7). Annotated genes with the strongest expression bias in the male brain included genes encoding neuropeptide precursors- galanin/GMAP prepropeptide (Gal), urotensin related peptide1 (Urp1), and CART prepropeptide; transmembrane receptors- glutamate receptors, hypocretin/orexin transmembrane receptor; Na^+^- K^+^- and Ca^2+^- cation transport channels; and lens crystallins- Crygm2d11 and Crygmxl2 (Figure 3B, Table 3A, Additional file 6: Table S4).

**Table 3:** **Enriched Gene Ontology (GO) terms in male-bias and female-bias genes**

GO term	SB (All)	p-value	Representative genes
**(A) Brain tissue**			
**Male: differentially expressed: 702; best** **Blastx** **hits in NR database: 420; genes with GO terms: 237**			
Ionotropic glutamate receptor signaling (GO:0035235: BP)	13 (46)	< 0.0001	GRIK5 (2of2); GRIK4; GRIN2A (1 of 2); Glutamate [NMDA] receptor subunit zeta-1
Ion transmembrane transport (GO:0034220: BP)	29 (529)	< 0.0001	Potassium channels: KCNJ3; KCNJ11 (1of2); KCNH1; KCNA1 (2 of2), Calcium channels: CACNA1I (4of4); Ryanodine receptor (RYR2 (1 of 3)), Sodium channel: SCN8A(2of2)
Regulation of cell development (GO:0060284: BP)	9 (203)	0.00699	Lens calpain-3 (CAPN3); distal-less homeobox gene-1a (DLX1a); protein tyrosine phosphatase receptor-d (PTPRD); retinal cadherin-4 (CDH4 (1 of 2))
Cerebellar Purkinje cell differentiation (GO:0021702: BP)	3 (11)	0.00068	LIM/homeobox protein (LHX1); voltage-dependent P/Q-type calcium channel subunit alpha-1A (CACNA1A) ; LIM domain binding-1 (LDB1(2 of 2))
Integral to membrane (GO:0016021: CC)	84 (2448)	< 0.0001	Hypocretin (orexin) receptor-1 (HCRTR1); multidrug resistance-associated protein 9; coiled-coil domain containing 149 CCDC149 (2 of 2); protocadherin 15b; gamma-aminobutyric acid A receptor, alpha-3 (GABRA3)
**Female: differentially expressed: 1764; best** **Blastx** **hits in NR database: 1596; genes with GO terms: 955**			
Endothelial cell migration (GO:0043542: BP)	10 (44)	< 0.00036	Sushi-repeat containing protein, X-linked2 (SRPX2); angiopoietin1 (ANGPT1); myosin heavy chain 9,non-muscle (MYH9(2 of 2))
Gonad development (GO:0008406: BP)	8 (37)	0.00194	WNT10A; WNT5A; secreted frizzled related proteins (SFRP1, SFRP5) ; TGFB2; Phospholipase A2 groupIVa (PLAG4A);
Immune response (GO:0006955: BP)	23 (135)	< 0.0001	WNT5A; TGFB2; kelch-like protein 6 (KLHL6); MHC classI-E (HLA-E); complement component (C6); MHC class II invariant chain (CD74(1of2)); chemokine(C-X-C motif) ligand12 (CXCL12(2of2))
Integrin-mediated signaling pathway (GO:0007229: BP)	8 (35)	0.00132	Integrin b1 binding protein1 (ITGB1BP1); Integrin, alpha10 (ITGA10);Integrin beta (ITGBL1, ITGB3a); nicotinamide riboside kinase-2 (NMRK2(2of2))
DNA-dependent DNA replication initiation (GO:0006270: BP)	5 (10)	0.0002	Minichromosome maintenance complex components (MCM2,MCM3,MCM4,MCM5,MCM6)
proteinaceous extracellular matrix (GO:0005578: CC)	61 (151)	< 0.0001	ADAM metallopeptidase with thrombospondin family members (ADAMTS12, ADAMTS15); NID1 (1of2); FBN3; MFAP2; CYR61 (2of2); COL4A1; COL10A1; COL11A1; WNT11; matrix metalloproteinases (MMP12, MMP14,MMP2 )
**(B) Tail tissue**			
**Male: differentially expressed: 755; best** **Blastx** **hits in NR database: 635; genes with GO terms: 404**			
Neuropeptide signaling pathway (GO:0007218: BP)	5 (15)	< 0.00016	Tachykinin, precursor 1 (TAC1); prepronociceptin PNOC (1 of 2); secretogranin V (7B2like) (SCG5); brain-specific angiogenesis inhibitor-3 (BAI3)
Neurotransmitter transport (GO:0006836: BP)	11 (46)	0.00057	Syntaxin1B (STX1B); solute carrier family (SLC6A2, SLC6A5); syntaxin binding protein 1 STXBP1 (1 of 2)
Locomotory behavior (GO:0007626: BP)	9 (42)	0.00072	Glycine receptor subunit beta (GLR-b2); choline O-acetyltransferase CHAT (2 of 2); astrotactin 1 (ASTN1)
*melanosome (GO:0042470:CC)	4 (15)	0.00241 0.01329	5,6-dihydroxyindole-2-carboxylic acid oxidase TYRP1 (1 of 2, 2 of 2); Tyrosinase TYR (2 of 2); synaptotagmin-like-2 (SYTL2); *adenosine deaminase CECR1*
*Pigment biosynthetic process (GO:0046148: BP)	4 (25)		
Insulin receptor binding (GO:0005158: MF)	4 (14)	0.00156	Sorbin and SH3 domain containing 1 SORBS1; DOK7 (1 of 2); growth factor receptor-bound protein 10 (GRB10)
**Female: differentially expressed: 705; best** **Blastx** **hits in NR database: 616; genes with GO terms: 387**			
Glycolysis (GO:0006096: BP)	12 (43)	< 0.0001	lactate dehydrogenase A (LDHA); phosphoglycerate kinase 1 (PGK1); pyruvate kinase, muscle (PKM (1 of 2)); glucose-6-phosphate isomerase (GPI (2 of 2)); 2,3-bisphosphoglycerate mutase (BPGM)
DNA-dependent DNA replication (GO:0006261: BP)	10 (29)	< 0.0001	Topoisomerase2a (TOP2a); primase, DNA, polypeptide 1 (PRIM1); tonsoku-like, DNA repair protein (TONSL); minichromosome maintenance complex components (MCM2, MCM4, MCM5, MCM6); polymerase (DNA directed), epsilon2 (POLE2)
Mitosis (GO:0007067: BP)	13 (91)	0.00105	Aurora kinase C AURKC; cyclin-dependent kinase 1 CDK1 ; spindle apparatus coiled-coil protein 1 SPDL1; cyclin B1 CCNB1; non-SMC condensin I complex, subunitH, subunitD2 (NCAPH, NCAPD2); checkpoint kinase-1 (CHEK1)
Proteinaceous extracellular matrix (GO:0005578: CC)	26 (125)	0.00106	Secreted protein, acidic, cysteine-rich (osteonectin) (SPARC); versican VCAN VCAN (2 of 2); tenascin(TNC (1 of 2)); collagens (COL11A1a, COL11A1b, COL27A1 (2 of 2))
**(C) Gonad Tissue**			
**Male: Differentially expressed: 4891; Best** **Blastx** **hits in NR database: 3879; Genes with GO terms: 2033**			
Cilium assembly (GO:0042384: BP)	32 (46)	< 0.0001	Radial spoke head 9 homolog (RSPH9); ARPC4-TTLL3 readthrough; forkhead box J1 (FOXJ1); Transmembrane proteins (TMEM237, TMEM17, TMEM231); B9 protein domain 1 (B9D1); coiled-coil and C2 domain containing-2A (CC2D2A)
Spermatogenesis (GO:0007283: BP)	25 (58)	< 0.0001	Kelch-like family member 10 KLHL10 (3 of 3); rhophilin associated tail protein 1-like (ROPN1L); phosphate cytidylyltransferase 1, choline-b ( PCYT1B (1 of 2)); Outer dense fiber protein 2/Cenexin (ODF2)
Meiosis I (GO:0007127: BP)	12 (25)	0.00025	MutS homolog 5 (E. coli) MSH5; DMC1 dosage suppressor of mck1 homolog, meiosis-specific homologous recombination (DMC1); HORMA domain containing 1 HORMAD1; cyclin B1 interacting protein 1, E3 ubiquitin protein ligase CCNB1IP1
Fertilization (GO:0009566: BP)	8 (14)	0.00064	KLHL10 (3 of 3); spectrin, beta, non-erythrocytic 4 SPTBN4 (1 of 2); glycine receptor, beta GLRB (1 of 2); polypyrimidine tract-binding protein-1 (PTPB1)
**Female: Differentially expressed: 5163; Best** **Blastx** **hits in NR database: 4577; Genes with GO terms: 2847**			
Regulation of BMP signaling pathway (GO:0030510: BP)	15 (27)	< 0.00028	Forkhead box H1 (FOXH1); Noggin (NOG); WNT5a; activin A receptor, type I (ACVR1); follistatin-like 1 (FSTL1); bone morphogenetic protein 15 (BMP15); growth differentiation factor 9 (Gdf9)
Fibroblast growth factor receptor signaling (GO:0008543: BP)	21 (47)	< 0.00094	Kinesin family member-16Bb (KIF16bb); sal-like-4 (SALL4); serine threonine-protein phosphatase 2a (PP2a); sal-like 1 (SALL1); catenin, beta 1 (CTNNB1); fibroblast growth factor receptor-1(FGFR1 (2 of 2)); sprouty homolog 1, antagonist of FGF signaling (SPRY1); FGF20, FGF13, FGF 10
Focal adhesion (GO:0005925: CC)	26 (50)	< 0.0001	Filamin A alpha FLNA (2 of 2); ezrin EZR (1 of 2); Rho guanine nucleotide exchange factor-7 (ARHGEF7 (al 3 paralogs)); syndecan-4 (SDC4); PDZ and LIM domain 2 (PDLIM2); talin 2 TLN2 (2 of 2)
Blood vessel development (GO:0001568: BP)	90 (234)	< 0.0001	Forkhead box H1 (FOXH1); lysophosphatidic acid receptor-2(LPAR2 (2 of 2)); angiopoietin-2 (ANGPT2); Rho guanine nucleotide exchange factor-7(ARHGEF7(3 of 3)); melanoma cell adhesion molecule (MCAM1 of 2); angiopoietin-like-1(ANGPTL1 (2 of 2)); activin A receptor, typeI (ACVR1); activin A receptor type II-like-1(ACVRL1)
Proteinaceous extracellular matrix (GO:0005578: CC)	50 (111)	< 0.0001	Netrin-4 (NTN4 (2 of 2)); sparc/osteonectin, cwcv and kazal-like domains proteoglycan (testican) (SPOCK2 (2 of 2)); collagens (COL9A2, COL5A2, COL11A1a, COL11A1b, COL27A1 (2 of 2)); ADAMTS8

For each tissue we show GO terms enriched in both male-biased and female-biased genes. We report the total number of genes that have sex-biased (SB) expression and the number of sex-biased genes that were annotated with Blastx against NR and with GO terms. For each enriched GO term we report the GO term, its ID, and ontology (BP, Biological Process; MF, Molecular Function, and CC, Cellular Component), the number of sex-biased sequences, number of expressed sequences (in brackets) and the p-value. Statistical significance values were calculated with the Fisher’s test using the elim algorithm for reducing comparisons. We also list representative genes associated with the enriched GO term ordered by fold change expression.

* indicates that the same genes (except the italicized gene) were associated with both the cellular component and biological process GO terms.

Most genes identified as female-biased in the brain were expressed in both sexes but with significantly higher expression in females (Figure 3A). These transcripts were enriched with GO terms related to cell migration, cell adhesion, development, DNA replication, growth, glycolysis, and immune response (Table 3A, Additional file 9: Table S7). Several top female-biased transcripts encoded components of the proteinaceous extracellular matrix. For instance, genes encoding nidogens, laminins, fibronectins, collagens, as well as specific matrix metalloproteinases (Mmp-2-14) and members of disintegrin and metalloproteinase with thrombospondin motifs (Adamts) family were higher expressed in female brain (Figure 3B, Table 3A). Annotated genes with the strongest expression bias in the female brain included genes encoding peptide hormones- growth hormone-1 (Gh1), chorionic gonadotrophin beta 1 (Cgb1) and prolactin (Prl); and the calcium binding proteins parvalbumin-2 (Pvalb2) and calsequestrin-1 (Casq1a). Expression of the gene encoding teleost brain-specific aromatase, cytochrome P450 19A1b (Cyp19a1b), was 5-fold higher in the female than the male brain (Figure 3B, Additional file 6: Table S4).

### Sex-biased gene expression in the tail

We found similar numbers of male-biased and female-biased genes in the tail (Table 2, Figure 3C). GO terms related to signaling pathways, vesicle organization and transport, and transmembrane transport were enriched in the male-biased sequences (Table 3B, Additional file 10: Table S8). Several of the top male-biased genes encoded proteins with functions in pigment biosynthesis (Figure 3D, Additional file 7: Table S5, see below for more detail). Among female-biased genes, GO terms for cell-division, DNA replication, repair and recombination, glycolysis, and extracellular matrix components were enriched (Table 3B, Additional file 10: Table S8). Differentially expressed genes with growth-related functions included genes encoding mitotic cell-cycle factors - cyclin B1, cyclin A2, cyclin dependent kinase-1, and mini-chromosome maintenance (MCM) replication initiation factors (Figure 3D, Table 3B).

**Figure 4:**
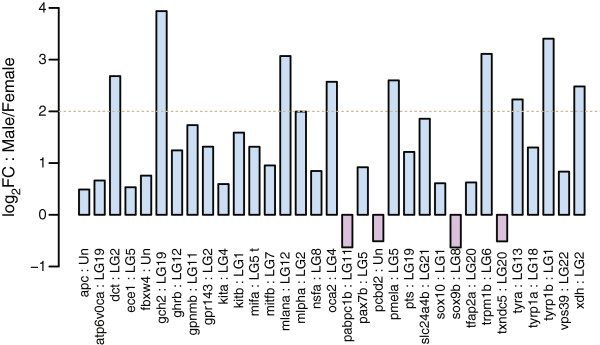
**Male-biased expression of guppy pigmentation orthologs in tail.** Barplots show male to female expression ratios (log_2_ FC: Male/Female) in tail tissue for differentially expressed candidate pigmentation genes (FDR < 0.1). Horizontal grey dotted line marks a 4-fold change in gene expression. Candidate gene names and linkage groups are specified at the bottom.

Adult male guppies display male-specific pigment patterns, therefore we examined differential expression of genes involved in pigmentation and patterning in more detail. We identified guppy orthologs for 132 genes and a few of their paralogs described for their role in pigment synthesis and pigment pattern formation in vertebrate model organisms (Additional file 13: Table S11) [51, 52]. None of these genes were identified as male-specific because all of these could be aligned to the assembled female genome. Of these pigmentation candidates, 33 genes showed significant differential expression between the sexes, with 29 showing male-biased expression (Figure 4). Among these, ten genes showed a four-fold higher expression in male tail tissue (encoding dopachrome tautomerase (Dct), GTP cyclohydrolase 2 (Gch2), melanoma A (Mlana), melanophilin A (Mlpha), oculocutaneous albinism II (Oca2), premelanosome a (Pmel-a)*,* transient receptor potential cation channel subfamily M member 1b (Trpm1b), tyrosinase a (Tyr-a), tyrosinase-related protein 1b (Tyrp1-b) and xanthine dehydrogenase (Xdh)).

### Testis-biased genes show high fold-change in expression

In gonads, 77% of all expressed genes showed sex-biased expression (Table 2, Figure 3E). We also found a number of genes with probable sex-limited expression in ovary or testis (Figure 3E). Male-limited and male-biased transcripts showed a greater magnitude of fold-change than the female-biased transcripts (Figure 3E, 3F). These included genes encoding some male-specific sex-development and differentiation associated proteins (e.g. DM-domain transcription factor Dmrt1, its paralog Dmrt2, and the 11-ketotestosterone biosynthesis enzyme Cyp11b2) (Additional file 8: Table S6); sperm associated antigens, ciliary and flagellar proteins (e.g., Spag17, Spag6, Tekt-1); spermatogenesis related - Spatc1l, Spata4; and testis expressed Tex9 (Figure 3F). Enriched GO-terms associated with male-biased genes included cilium assembly, spermatogenesis, and microtubule-based movement (Table 3C, Additional file 11: Table S9). Among the female-limited and female-biased sequences, we found genes encoding aromatase A (Cyp19a1a), the zona pellucida glycoproteins Zp1 and Zp2, oocyte specific proteins Zar1 and Zar1l (Figure 3F), ovarian folliculogenesis factors Gdf9 and Bmp15, and forkhead domain transcription factors Foxl2 and Foxr1 (Table 3C, Additional file 8: Table S9). Over-represented GO terms associated with female-biased genes were blood vessel development, regulation of BMP signaling pathway, amino acid transport, focal adhesion, cell migration involved in gastrulation, FGF receptor signaling, apical protein localization, regulation of body-fluid levels, and gas transport (Tables 3E, Additional file 11: Table S9).

### Genes with common sex-biased expression in multiple tissues

A greater number of female-biased than male-biased genes showed a common direction of sex-bias in two or all three tissues (Table 2). Over-represented GO terms among genes with female-biased expression in both brain and tail included glycolysis, DNA replication and recombination as biological process terms, and proteinaceous extracellular matrix as cellular component term. While only a few genes showed male-biased expression in both brain and tail, we found that enriched terms related to transmembrane ion transport were common to both (Tables 3A, B, Additional file 12: Table S10).

### Non-random distribution of sex-biased genes on the female genome

Based on alignment positions of all genes on the currently available female draft genome sequence, we analyzed the distribution of all sex-biased genes (1.2 fold; FDR < 0.05) in comparison to all expressed genes (log_2_CPM > 2) along the guppy linkage groups. The total number of sex-biased genes per chromosome with their observed proportions and significance values for difference from expected proportions is described in Additional file 14: Table S12.

**Figure 5:**
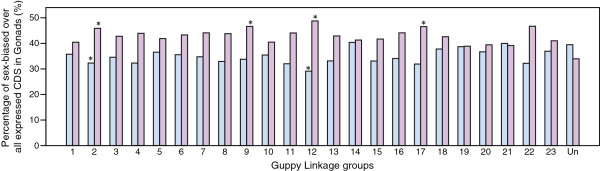
**Linkage group distributions of sex-biased genes.** Distribution of percentage of testis-biased (blue) and ovary-biased (pink) genes over all gonad-expressed genes per linkage group (LG). Sex-biased genes were identified as those that show significant difference in expression (FDR < 0.05) above the 1.2 fold-change (log_2_FC: Male/Female). LGs with a significant over- or under-representation of sex-biased genes are marked with an asterisk (p < 0.05, after multiple correction).

We found that the frequencies of ovary-biased genes on LG2, 9, 12 and 17 and testis-biased genes on LG2,12, is significantly different (p < 0.05, corrected for multiple testing) from the frequencies expected for a random distribution of sex-biased genes in the gonads (Table 4, Figure 5). Among these, the greatest difference was seen for LG12, the putative X-chromosome [45, 53], where we found 26% greater than expected proportion of ovary-biased genes and 23% less than expected proportion of testis-biased genes (Figure 5, Additional file 14: Table S12). Although, we found a higher proportion of ovary-biased genes and lower proportion of testis-biased genes on LG2, 12, 17, and 22, only LG2 and LG12 show a significant difference after correction for multiple testing (Table 4). Sex-biased genes in the somatic tissues brain and tail did not show significant non-random distribution on any linkage group. Higher fold-change in expression suggests greater sex-specificity; we therefore repeated our analysis counting all genes that showed median-fold differential expression between sexes (FDR < 0.1). We found accumulation of genes with 3.2-fold higher expression in ovary than testis on LG12 and LG17 but the difference was not significant after correcting for multiple comparisons (Table 4). For genes with median-fold sex biased expression in somatic tissues, a significantly greater proportion of male-biased genes in brain and lower proportion of female-biased genes in gonads (p < 0.05, corrected for multiple testing) was found on the scaffolds that could not be assigned to any linkage group (Additional file 14: Table S12).

**Table 4:** **Linkage groups (LGs) with over-representation or under-representation of male-biased or female-biased genes**

	Male-biased genes over represented	Male-biased genes under represented	Female-biased genes over represented	Female-biased genes under represented
***All***				
Brain				LG10
Tail		LG16		
Gonad		**LG2,** LG4, LG8, LG11, **LG12**, LG15, LG17, LG22	**LG2, LG9, LG12**, **LG17,** LG22	
***Median***				
Brain	LG5	LG21		
Tail	LG17			
Gonad		LG4, LG11	LG12, LG17	

We use two expression differences cut-offs, ‘All’, 1.2-fold difference in expression (FDR < 0.05) and ‘median’, median-fold difference in expression (FDR < 0.1) for three different tissues (brain, tail, and gonad). The LGs with significant over- or under-representation of sex-biased genes (p < 0.05) are listed and those with p < 0.05 after correcting for multiple testing are highlighted in bold.

### Rapid evolution in sex-biased genes

**Figure 6:**
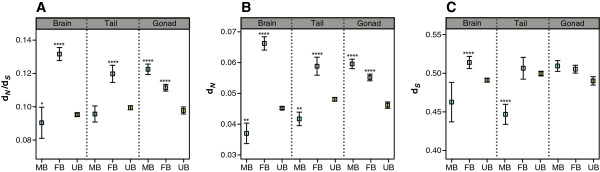
**Nucleotide substitution rates in sex-bias genes per tissue.** Mean values with 95% confidence intervals for rate of nucleotide substitutions in coding sequences. **(A)** *d*_*N*_ */d*_*S*_ ratios; **(B)** *d*_*N*_; and **(C)** *d*_*S*_. Male-biased (MB: blue), female-biased (FB: pink), and unbiased (UB: yellow) genes for brain, gonad, and tail. Asterisks above the boxplots indicate a significant difference in substitution rate was found between the sex-biased and unbiased genes using Mann–Whitney U test for non-parametric distributions (**** p < 0.0001; *** p < 0.001; ** p <0.01; * p < 0.05).

Using ProteinOrtho we obtained 12,801 single-copy orthologous protein sequences, between guppy, medaka and stickleback. After estimating rates of non-synonymous (*d*_*N*_) and synonymous (*d*_*S*_) substitutions, and rejecting sequences with high *d*_*S*_, 11,124 three-way alignments (1:1:1 orthologs) remained. The genes with female-biased expression in the brain had higher mean values for *d*_*N*_*/d*_*S*_ and *d*_*N*_ compared to unbiased and male-biased genes. Similarly, we observed higher mean values for *d*_*N*_*/d*_*S*_ and *d*_*N*_ of genes with female-biased expression in the tail, but the difference between means was not as high as found in the brain (Figure 6, Table 5). In gonad tissues, both the testis-biased genes and ovary-biased genes had a significantly higher average *d*_*N*_*/d*_*S*_ and *d*_*N*_ than the unbiased genes.

**Table 5:** **Comparison of d**_**N**_ **/d**_**S**_ **values for sex-biased genes to unbiased genes for brain, tail, and gonad tissues**

	Brain			Tail			Gonad		
	(N)	d_N_/d_S_	p-value	(N)	d_N_/d_S_	p-value	(N)	d_N_/d_S_	p-value
***All***									
Unbiased	7386	0.097		5466	0.100		1667	0.099	
Male-biased	105	0.082	0.015	279	0.096	0.474	1350	0.122	**< 0.0001**
Female-biased	653	0.129	**< 0.0001**	266	0.119	**< 0.0001**	1984	0.112	**< 0.0001**
***Autosomal***									
Unbiased	6061	0.096		4383	0.100		1328	0.097	
Male-biased	96	0.084	0.083	221	0.094	0.576	1111	0.122	**< 0.0001**
Female-biased	542	0.131	**< 0.0001**	199	0.119	**< 0.0001**	1561	0.111	**< 0.0001**
***Single Tissue***									
Unbiased	2018	0.094		504	0.124		104	0.135	**(< 0.0001)**
Male-biased	74	0.077	0.061	179	0.091	**0.00014**	1070	0.122	**0.0038 (< 0.0001)**
Female-biased	310	0.130	**< 0.0001**	98	0.122	0.337	1368	0.108	**0.00014 (< 0.0001)**
***Multiple Tissue***									
Unbiased	4043	0.096		3879	0.097		1224	0.094	
Male-biased	22	0.107	0.929	42	0.107	0.452	41	0.105	0.336
Female-biased	232	0.132	**< 0.0001**	101	0.116	**0.0017**	193	0.131	**< 0.0001**
***Highly expressed***									
Unbiased	2220	0.079		1103	0.090		550	0.084	
Male-biased	23	0.076	0.328	50	0.102	0.120	684	0.125	**< 0.0001**
Female-biased	183	0.115	**< 0.0001**	40	0.104	0.191	499	0.112	**< 0.0001**

We first compare mean d_N_/d_S_ values for all sex-biased genes (FDR < 0.1 and median fold-change: brain 1.5 fold; tail 1.7 fold; gonad 3.5 fold) to unbiased genes (FDR > 0.1, log_2_CPM > 2). We then compare sex-biased genes mapped to autosomal linkage groups only. We also compare those expressed only in a single tissue and multiple tissues separately. In the single tissue gonad set we also compared d_N_/d_S_ to unbiased genes on autosomal linkage groups only (p-values given in brackets). Finally, we compared only highly expressed genes (>32 CPM). For each comparison we give the number of genes (N), the mean d_N_/d_S,_ and p-value, with p-values less than 0.0001 in bold.

Sex-linked genes may evolve at faster rates due to recombination differences between male and female germline, we therefore repeated the analysis using only autosomal genes and found similar rates of coding sequence evolution (Table 5). Also, magnitude and breadth of gene expression may be associated with functional constraints on coding sequence evolution [54]; we therefore further compared autosomal genes that were sex-biased in single tissues, multiple tissues, and had overall high expression (log_2_CPM > 5 i.e. 32 counts per million). Grouping by expression level or by expression breadth did not change the trend for higher *d*_*N*_*/d*_*S*_ of genes with female-biased expression in brain or sex-biased expression in the gonads (Table 5).

## Discussion

### Assembly of a reference transcriptome

Recent studies have shown that different assembly algorithms have varying strengths and limitations and a comprehensive assembly strategy should include the use of multiple assemblers [55–57]. While information from genomic coordinates assists in the assembly of full-length transcripts, at the same time genome-independent assemblies are free from biases caused by potential gaps and mal-orientations found in draft genomes. Therefore, we combined both assemblies to generate a non-redundant reference transcriptome composed of 74,000 CDS. Approximately, 24,000 CDS (~35%) were assigned as *bona fide* orthologs of published coding sequences. The remaining sequences may represent partially assembled sequences as well as incomplete CDS predictions and they may also include as yet unknown CDS (e.g., novel paralogs or splice variants), noncoding RNA, or assembly artifacts like chimeric transcripts. Our reference transcriptome provides a resource for investigating the genetics of complex adaptive traits such as guppy color patterns, life-history, and behavior [32, 38, 58].

### Sex-biased gene expression associated with phenotypic dimorphism

Based on GO terms and orthology predictions, we attempted to relate our observations on sex-biased gene expression to sexually dimorphic phenotypic traits of the guppy. Pigment cells contributing to the adult male ornaments were expected to show a sex-biased expression mainly in adult skin, included as part of the tail in this analysis. Of the candidate genes associated with pigmentation, several were indeed higher expressed in male tails. A distinctive trait of the live-bearing female guppies is their lifelong growth, while male growth slows down after puberty [32, 59]. In concordance with this phenotypic difference, transcripts encoding cyclins and kinases characteristic of the mitotic phase of the cell cycle, DNA replication proteins and several growth factors were higher expressed in the female tail.

Female-biased expression of genes encoding cell-cycle and growth related hormones was also observed in the brain. Moreover, transcripts of the neurogenic zone associated aromatase, cyp19a1b, were highly expressed in the female brain but not the male brain, suggesting sexual dimorphism in adult neurogenesis in the guppy [60, 61]. We found a female bias in expression of many ECM components, which previously have been associated with neurogenesis and synaptic plasticity [62, 63]. Interestingly, greater brain plasticity in females as compared to male guppies has been postulated based on predator avoidance, kin-recognition, and mate choice differences in the wild [38, 42, 64, 65]. We also detected male-biased transcripts that encode neuropeptides and several transmembrane receptors in the brain, suggesting sex-differences in signal transduction. One example of such a male-biased transcript encodes the neuropeptide galanin, known to be involved in the neuroendocrine regulation of growth and reproduction in fish [66]. Galanin neuropeptide and its receptor have also been shown to be more highly expressed in parts of the male versus female brain of sailfin mollies (*Poecilia latipinna*) [67, 68].

The highest degree of sex-bias in gene expression was found in the gonads, as expected in a gonochoristic organism. Expectedly, testis-biased transcripts encoded proteins with functions pertinent to testicular cells, e.g. spermatogenesis, sperm motility, and meiosis. Testis-specific and testis-biased expression of genes encoding Dmrt1 and Dmrt2, respectively, suggests a requirement of these transcription factors for testis maintenance [69]. Ovary-biased transcription factors included the steroidogenesis regulators Foxl2 and Nr5a1 [70]. Continuous FOXL2 activity is known to be required for suppression of trans-differentiation of ovarian cells into testicular cells in adult mice [71, 72]. The ovary-specific expression of the aromatase Cyp19a1a and testis-biased expression of Cyp11b2*,* which encodes for an enzyme for androgen 11-ketotestosterone biosynthesis [70], also indicates differences in sex-steroid synthesis in the two tissues. According to functional GO classification, female-biased genes were enriched for follicular vascularization factors, likely related to the lecithotrophic developmental strategy of the guppy [73]. During oocyte growth in lecithotrophic species, the follicle is required for efficient transport of yolk precursors and probably amino acids and other metabolites from the blood to the maturing oocyte. After fertilization, the highly vascularized follicle persists as a placenta that is essential for osmoregulation, gas exchange, waste disposal, and transport of some essential factors [74, 75].

### Chromosomal distribution of sex-biased genes

We found a significant enrichment of female-biased genes on the putative X-chromosome, LG12. Sex-biased genes have been reported to accumulate on differentiated sex-chromosomes of many species. Enrichment of female-biased genes on X-chromosomes has been reported in species with heterogamous males, e.g. several *Drosophila* species [21, 76], mouse [77], and the nascent X-chromosome of the stickleback (*Gasterosteus aculeatus*) [22]. Similarly, enrichment of male-biased genes has been found on the Z-chromosomes of species with heterogamous females, e.g. zebra finch (*Taeniopygia guttata*) and chicken [20]. In guppies, the majority of the sex chromosome is pseudo-autosomal, yet the X and Y chromosome show genetic and cytological distinctions [54, 78]. Although differentiation between X- and Y-chromosomes is not comparable to the situation in mammals, birds, or drosophilids, the over-representation of ovary-biased genes and under-representation of testis-biased genes on the guppy LG12 indicates sex-specific selection pressures even in the absence of a truly hemizygous state in males. Previous studies have indicated reduced synaptic pairing [78] and reduced recombination [54] between X- and Y-chromosomes during male gametogenesis in guppies. This may lead to accumulation of deleterious mutations in Y-linked alleles of genes on LG12. Ovary-biased or ovary-specific genes are likely not needed in males and therefore mutations in these genes will persist on the Y-chromosome, while mutations in testis-biased genes and other non-biased genes will be selected against. Further analysis of recombination frequencies and gene order along the length of the sex chromosomes, coupled with comparisons across multiple populations, will enable better understanding of the effect of genomic location of sex-biased genes.

### Molecular evolution of sex-biased genes

Our comparisons of *d*_*N*_*/d*_*S*_ of sex-biased and unbiased genes in the guppy revealed elevated coding sequence change of testis and ovary biased genes, and female-biased genes expressed in the brain and those co-expressed with a female-bias in the brain and at least one of the other tissues. Current knowledge on protein evolution suggests that sex-biased genes in reproductive and non-reproductive tissues show accelerated evolution in many species including *Drosophila*, mouse, and chicken [18, 19, 79, 80]. Sex-biased genes may show rapid divergence due to their evolution under sexual selection. Additionally, accelerated sequence divergence may also occur under sex-specific natural selection, or relaxed purifying selection on genes that have reduced functional pleiotropy [15, 81]. Our results indicating rapid evolution of sex-biased genes in gonads driven by testis-biased genes are in concordance with the results obtained from other vertebrates [18, 82, 83]. Rapid divergence of reproductive proteins driven by testis-specific proteins may be involved in sexually-antagonistic selection [84], post-copulatory sexual selection resulting from co-adaptation [85], or kinship recognition and incipient speciation in guppies [86]. These processes may be important in guppies given their highly promiscuous mating system with coercive internal fertilization by males and long-term sperm storage in females [39].

We also found a higher *d*_*N*_*/d*_*S*_ ratio in female-biased genes expressed in the brain. While considerable evidence suggests that sexual selection in guppies is driven by the multivariate mating preferences of females, male-male competition, male mating tactics, and male mate choice may also be under selection [37, 38, 87, 88]. An association between molecular evolution of female-biased genes and sexual selection on behavioral traits was not easy to elucidate as these genes were expressed in both sexes and co-expressed in multiple tissues, suggesting some pleiotropy in function. Rapid evolution of female-biased genes with growth-related and metabolic functions may be pertinent to the sexual size dimorphism seen in this species and may be driven by natural selection on life-history traits [89].

Unexpectedly, we also found signatures of rapid evolution in female-biased genes whose expression was not tissue-specific. Usually a broad expression profile indicates pleiotropic functions that would constrain divergence of coding genes [19, 90]. This prediction is, however, not necessarily cogent for fish, where the teleost-specific whole genome duplication allows for evolving sub-functionalization or even some redundancy when co-orthologs are preserved [91]. Furthermore, many of the co-expressed female-biased genes identified in our study encode ECM components, cell-cycle factors, and glycolytic enzymes. These proteins have conserved functions across all tissues and therefore may not be pleiotropic. Moreover most of these proteins are located in the cytosol or in the extracellular region where adaptive evolution has been described to be more relaxed [90, 91]. Conversely, no difference in evolutionary rate was found between male-biased genes co-expressed in the brain and tail and unbiased genes. Many of these genes encoded neuropeptides, transmembrane receptors, and gated ion-channels that evolve under structural and functional constraints of ligand-receptor specificity and transmembrane localization [90]. Therefore, these proteins are likely to have low tolerance of mutations and more conserved evolution.

## Conclusion

Our analyses of sex-biased gene expression in guppies revealed differences that are likely to be pertinent for the mechanisms underlying its sexual dimorphism. The observed female-biased expression of growth-related genes in the three tissues investigated could reflect the life-long growth observed in female guppies. At the same time, elevated male-biased expression of genes known to be relevant for pigment cell differentiation was limited to the tail, the tissue including part of the adult skin. As expected, sperm-specific and cell-cycle-relevant transcripts were highly expressed in the testis, and the expression profile of the ovary signifies maternal provisioning of this lecithotrophic species. Correlations between gene expression and phenotypic traits will remain speculative in guppies until methods of experimental gene gain and loss of function can eventually be established in this system.

We detected accumulation of ovary-biased genes on the putative X-chromosome, supporting the hypothesis that genes advantageous to only one sex accumulate on the differentiating sex chromosomes. We also observed more rapid evolution of testis-biased genes, possibly indicating increased sexual selection on males. However, the observed rapid evolution of genes with female-biased expression in brain and other tissues not seen in males may be confounded by differences in the biological functions and cellular locations of male- and female-biased genes. It is probable then, that there are differences in selection on protein sequences of males and females independent of the breadth of tissue expression.

## Methods

### Fish strains, husbandry, and dissection

This study was carried out in accordance with the German Protection of Animals Act (§ 11 Abs. 1 Nr. 1 a und b TierSchG). All animal experiments were permitted by the Regierungspräsidium Tübingen (approval ID 35/9185.46). Sample tissues were prepared from laboratory-reared guppies that were descendants of wild fish caught in 2003 from a low-predation population in Quare river, East Trinidad [45, 92]. The fish were reared under uniform conditions of food, water, light, and density. Mature adult guppies between 5–6 months of age were isolated and kept without food in fungicide treated water for 44–48 hours prior to dissections. Fish were euthanized using 0.1% (w/v) tricaine (ethyl 3-aminobenzoate methanesulfonate salt) solution pH 7. Brain, eyes, liver, spleen, skin, tail (the post-anal tissue up to the beginning of the tail fin, containing adult skin, skeletal muscle, dorsal cord, bone, and cartilage), and gonads were isolated from euthanized adult males and females. Female tissues were prepared from virgin adults that were separated from males at the age of 3–4 weeks to avoid pregnancy and sperm storage. Whole embryos at late-eyed to very late-eyed stages of development [93] were isolated from gravid females. A small fin-clip was taken from each embryo for genotyping the sexes. All samples were washed with cold PBS (kept at 4°C), then frozen in liquid nitrogen, and stored at −80°C.

### Library preparation and sequencing

#### Non-barcoded libraries

Four Illumina cDNA libraries were prepared separately from female and male late-eyed stage embryos, and adult female and male tissues (brain, eyes, liver, spleen, skin, tail, and gonad tissues). Embryos were first genotyped using genomic DNA isolated from fin-clips and markers 229 and 230 with sex-specific single nucleotide polymorphisms (SNPs) in Quare population [54].

All tissue samples were homogenized in TRIzol® reagent (Invitrogen) using a Polytron® homogenizer (PT 1200, Kinematica AG, Switzerland) and total RNA was extracted from the Trizol homogenate according to manufacturer’s instructions. After removal of contaminant DNA, using DNaseI (Invitrogen), purified RNA was quality-checked and quantified (Nanodrop ND-2000, ThermoScientific peqlab®). For male and female adult libraries, a total of 75 μg RNA was prepared by pooling 15 μg of RNA isolated from each tissue. For male and female embryo libraries, 75 μg total RNA was isolated from 15 individual embryos each. Then purified polyA + mRNA (Dynabeads® Oligo(dT), Invitrogen) was used for preparation of paired-end RNA libraries with 200-300 bp insert size, using the mRNA-seq Sample Preparation Kit (Illumina, San Diego, CA) or the NEBNext® mRNA Library Prep Reagent Set for Illumina (NEB), according to manufacturer’s instructions. Library quality and concentrations were assessed using the Agilent DNA 1000 Bioanalyser Assay (Agilent Technologies, Germany). Each library was sequenced on a separate GAIIx lane (Illumina, San Diego, CA, read length 101 bp). These four datasets are referred to as female and male adult (F_adult_, M_adult_) and female and male embryo (F_embryo_, M_embryo_; Figure 1).

#### Barcoded libraries

Barcoded cDNA libraries were prepared for quantitative analysis of gene expression differences. Tissues from adult male and female brain and eyes, tail, and gonads (ovaries from virgin females or testes from males) were isolated as indicated in Figure 2. All tissues were individually homogenized using steel beads for disruption in TRIzol Reagent (Invitrogen, Carlsbad, CA, USA). Total RNA was extracted from the TRIzol homogenate using DirectZol RNA extraction kits with in-column DNaseI treatment. Purified total RNA was quality-checked on agarose gels and quantified using the Qubit RNA Assay Kit (Invitrogen, Carlsbad, CA, USA). Six biological replicates were prepared for each tissue and sex, except the female brain, which consisted of 7 biological replicates and two technical replicates (but only 6 biological replicates were used for quantitative comparisons). All samples were randomized and individually barcoded during library preparation using TruSeq mRNA-seq Sample Prep Kit (Illumina, San Diego, CA, mRNA-seq Sample Prep Manual v2 protocol). In total, 39 paired-end libraries were prepared and sequenced on 3 lanes of the HiSeq™ 2000 (Illumina, San Diego, CA, read length 101 bp, 13 libraries per lane). The barcoded cDNA libraries from adult tissues are referred to as: F_brain_, M_brain_, F_tail_, M_tail_, F_gonad_, M_gonad_ (Figures 1 and 2).

The types of tissues, number of individuals, types of libraries and sequence datasets are summarized in Additional file 1: Table S1.

### Quality filter and read trimming

The resulting reads in the non-barcoded datasets were filtered for low complexity using SHORE v0.6 [94] and for PCR duplicates by removing read pairs that matched 60 bp of both reads of a pair and keeping unique pairs and 3 duplicates with highest quality scores (customized perl script). We trimmed homopolymer sequences (polyA/T/G/C) over 22 bp length using Cutadapt v1.2.1 [95] and low-quality nucleotides using CONDETRI v2.2 [96] with a phred20 quality cutoff, 35 bp length cutoff and other default parameters. The barcoded dataset used in expression analysis was filtered only for low quality but trimmed similarly.

### Transcriptome assembly

A genome-independent transcriptome was assembled using Trinity (trinityrnaseq_r2012-06-08) [97] with minimum k-mer coverage of 2 and default parameters. In order to maximize the k-mer overlap and to achieve high coverage for rare transcripts, we combined all datasets (F_adult_ + M_adult_ + F_embryo_ + M_embryo_ + F_brain_ + M_brain_ + F_tail_ + M_tail_ + F_gonad_ + M_gonad_) and assembled a single *de novo* reference trancriptome with Trinity (Figure 1).

A genome-guided transcriptome assembly was compiled using TOPHAT – CUFFLINKS – CUFFMERGE v2.0.4 [98, 99] with default parameters using a draft assembly of the female guppy genome (Künstner *et al*., in preparation). Reads from each RNA-seq sample were first individually mapped to the reference genome using TOPHAT2 to retain tissue-specific splicing information. The resulting alignment files were analyzed by Cufflinks to generate a transcriptome assembly for each dataset (F_adult_, M_adult_, F_embryo_, M_embryo_, F_brain_, M_brain_, F_tail_, M_tail_, F_gonad_, and M_gonad_). These assemblies were then merged to give a combined assembly with CUFFMERGE (Figure 1).

### ORF prediction with TRANSDECODER

Open reading frames (ORFs) were predicted using the program TransDecoder from Trinity. Predicted coding sequences (CDS) over 150 bp long were clustered if they were greater than 90% similar and the longest sequence was kept in a non-redundant database using Cd-Hit-Est v4.6 [100, 101].

### Identification of orthologous proteins in other vertebrates

Orthologous genes to other vertebrate species were identified using translated CDS for both genome-guided and genome-independent assemblies. Sequences from *Danio rerio* (zebrafish), *Gasterosteus aculeatus* (stickleback), *Oryzias latipes* (medaka), *Xiphophorus maculatus* (platyfish), *Oreochromis niloticus* (tilapia), *Takifugu rubripes* (fugu), *Tetraodon nigriviridus* (tetraodon), *Gadus morhua* (cod), *Homo sapiens* (human), and *Mus musculus* (mouse) were downloaded from Ensembl (Release 71) [102]. Single-copy (1:1) orthologs were identified using ProteinOrtho v4.26 [103] (parameters: NCBI Blastp[104]] v2.2.21, E-value <1 × 10^−10^, alignment connectivity: 0.8, coverage: 40%, identity: 30%, adaptive similarity: 0.95, including pairs: 1).

### Alignment of reads to different assemblies

Pooled paired-end reads from all sequenced datasets were normalized using Diginorm[105] with default parameters for single-pass normalization. Reads from the normalized dataset were aligned to the genome-guided and the genome-independent assemblies using BOWTIE2 v2.0.04 [106] (default parameters for sensitive local alignment).

### Merged reference transcriptome and functional GO annotation

We merged the genome-independent and genome-guided assemblies by pooling the predicted CDS from both assemblies followed by clustering sequences with greater than 90% identity using Cd-Hit-Est to create a guppy reference transcriptome (GRT).

Annotations were found using NCBI BlastX v2.2.25 and the NCBI non-redundant protein database [50]. Functional categories were assigned by mapping GO terms using Blast2GO® v2.7.0 [107]. For simplicity we refer to the predicted CDS of the guppy reference transcriptome as genes in the text.

### Alignment against female genome

Genomic coordinates of genes in the reference transcriptome were obtained by aligning them against the repeat-masked draft female genome using GMAP v2012-07-20 [108]. In the case of ambiguous alignments, the alignments with the highest total coverage and identity were kept (total 607).

### Differential expression analysis

Each barcoded sequenced library from the organ datasets (F_brain_, M_brain_, F_tail_, M_tail_, F_gonad_, M_gonad_) was individually aligned to genes of the guppy reference transcriptome using BOWTIE2 v2.0.04. Mapped reads were counted using eXpress v1.3.1 [109]. Read counts from six individually barcoded biological replicates per tissue were used for differential expression analysis between male and female tissues using the Bionconductor[110] package edgeR v3.0.8 [111]. First low abundance genes with less than two counts per million mapped reads (<2 CPM/sample) across six samples were removed. Read counts were normalized for sequencing depth using TMM normalization [112] and differential expression between sexes was tested with a modified exact test implemented in edgeR [113] and corrected for multiple testing. Genes with significant expression difference between the sexes (FDR < 0.1 or if mentioned FDR < 0.05) were classified as sex-biased and those without a significant difference as ‘unbiased’. Using an FDR cut-off of 0.1 sex-biased sequences showed at least a 1.2 fold difference (log_2_FC > 0.3 or < −0.3) in expression between the sexes. Genes with sex-specific functions may have varying levels of expression divergence in different tissues [12, 19]. Therefore, we further categorized the sex-biased genes by fold-change, keeping genes with greater than median-fold difference in expression between sexes (log_2_FC: Male/Female) in each study tissue. These median-fold cutoffs were: 1.5 fold in the brain (log_2_FC > 0.6 or < −0.6); 1.7 fold in the tail (log_2_FC > 0.8 or < −0.8); and 3.2 fold in the gonad (log_2_FC > 1.8 or < −1.8). Enrichment of GO terms between sex-biased and unbiased genes per tissue was determined using a Fisher’s exact test with the Elim algorithm (p < 0.01, and number of sequences > 3) in the R package: topGO v2.10.0 [114].

### Chromosomal distribution of sex-biased genes

Non-random chromosomal distribution of male- or female-biased genes expressed in a tissue was tested with a χ^2^-test. P-values were corrected for multiple testing using the Benjamini-Hochberg method [115]. The expected distribution was calculated by assuming that sex-biased genes are randomly distributed across chromosomes and that their representation on a particular chromosome is proportional to the number of expressed genes on that chromosome. In the brain the average number of male-biased genes was significantly lower than the average number of female-biased ones; therefore for this tissue we calculated the expected frequency of male- and female-biased genes using their respective averages. We conducted this analysis twice, (1) where sex-bias was defined as a greater than the 1.2-fold-change between the sexes (FDR < 0.05) and (2) where sex-bias was defined as greater than median fold difference (FDR < 0.1). All comparisons were tested using statistical tests implemented in R package Stats version 2.15.2 [116].

### Alignment and evolutionary analysis

Orthologous amino acid sequences between stickleback, medaka, and guppy obtained from ProteinOrtho were aligned using Mafft v7.017b [117] and back-translated to nucleotide sequences for subsequent analysis. All alignments are available upon request. Substitution rates were estimated separately for synonymous (*d*_*S*_) and non-synonymous (*d*_*N*_) substitutions using a maximum likelihood method implemented in the Codeml program (model = 1, user tree specified according to the phylogeny) in the Paml package v4.1 [118]. We excluded all alignments shorter than 150 bp or with *d*_*S*_ larger than 2 to minimize statistical artifacts from short sequences and saturation effects in *d*_*S*_. Mean values of *d*_*N*_*/d*_*S*_ of male-biased and female-biased genes were compared to unbiased genes with significant expression (log_2_CPM > 2) per tissue.

All comparisons were tested using the non-parametric Mann–Whitney test as well as permutation tests with 1000 replicates (data not shown) using R version 2.15.2 [116].

### Availability of supporting data

The RNA-seq reads reported in this study have been deposited in the National Center for Biotechnology Information Short Reads Archive, http://www.ncbi.nlm.nih.gov/sra (Study Accession ID: SRP033586). The predicted CDS of the guppy reference transcriptome are available from our institute’s website: ftp://ftp.tuebingen.mpg.de/ebio/publication_data/esharma/guppy_trans/trin_cuff_v14_cdhit90.fa.gz

## Electronic supplementary material

Additional file 1: Table S1: Description of Illumina cDNA libraries - sample preparation and sequenced datasets. (DOCX 27 KB)

Additional file 2: Table S2: Best Blastx hits of the guppy reference transcriptome. Table shows guppy reference query with best–hit identified against NCBI non-redundant (NR) protein database (E-value < 1 x 10^−15^). (XLSX 4 MB)

Additional file 3: Table S3: Gene Ontology identities (GO IDs) annotated to coding sequences from guppy reference transcriptome. Sequences with a match in NR database (E-value < 1 x 10^−15^) were annotated as implemented in Blast2GO. (XLSX 635 KB)

Additional file 4: Figure S1: Spearman’s correlation in expression. Hierarchical clustering of Spearman rank correlations. Coloring indicates spearman’s correlation in gene expression between samples from barcoded datasets **(A)** and all datasets **(B)**. The dendrogram shows the agglomerative clustering (Ward’s) with the bootstrap values (percentage) showing the confidence in each branch. All samples show organ-specific clustering except for the gonads, that are most distinct from all other organs and cluster by sex. Female Brain FB; Male Brain MB; Female tail FT; Male tail MT; Female gonad FG; Male gonad MG; Female adult FAD; Male adult MAD; Female embryo FEM; Male embryo MEM. (PDF 471 KB)

Additional file 5: Figure S2: Expression differences between sexes. Expression differences between sexes. Distribution of expression statistics for genes with male-biased (MB), female-biased (FB), and unbiased (UB) expression in brain (grey), tail (yellow) and gonads (blue). **(A)** Boxplots show distribution of log_2_FC (Fold change: Male/Female). The lower median of each pair was used as cut-off for significant fold-change for that comparison (brain = 0.6; tail = 0.8; gonad = 1.8); **(B)** Boxplots show distribution of log_2_CPM (Counts per million) for sex-biased genes in each tissue pair. **(C)** Boxplots show distribution of coefficient of variation (CV) for all sex-biased genes (FDR <0.1) with greater than 1.2-fold change in expression and all unbiased genes (FDR > 0.1). **(D)** Boxplots show distribution of coefficient of variation (CV) for sex-biased genes (FDR <0.1) with greater than median-fold change in expression and all unbiased genes (FDR > 0.1). Outliers in Figure C and D are shown with black points. (PDF 3 MB)

Additional file 6: Table S4: Genes with sex-biased expression in brain. Sex-biased genes (FDR < 0.1) are shown with expression values and gene annotations (if available) obtained from annotated orthologs in other vertebrates. (XLSX 1003 KB)

Additional file 7: Table S5: Genes with sex-biased expression in tail. Sex-biased genes (FDR < 0.1) are shown with expression values and gene annotations (if available) obtained from annotated orthologs in other vertebrates. (XLSX 862 KB)

Additional file 8: Table S6: Genes with sex-biased expression in gonads. Sex-biased genes (FDR < 0.1) are shown with expression values and gene annotations (if available) obtained from annotated orthologs in other vertebrates. (XLSX 5 MB)

Additional file 9: Table S7: Over-represented Gene Ontology (GO) categories for sex-biased genes in brain. GO terms that were over-represented (p <0.01, No. of sequences > 3) among median-fold sex-biased genes as compared to all expressed genes in the brain are described. (XLSX 18 KB)

Additional file 10: Table S8: Over-represented Gene Ontology (GO) categories for sex-biased genes in tail. GO terms that were over-represented (p <0.01, No. of sequences > 3) among median-fold sex-biased genes as compared to all expressed genes in the tail are described. (XLSX 17 KB)

Additional file 11: Table S9: Over-represented Gene Ontology (GO) categories for sex-biased genes in gonads. GO terms that were over-represented (p <0.01, No. of sequences > 3) among median-fold sex-biased genes as compared to all expressed genes in the gonads are described. (XLSX 18 KB)

Additional file 12: Table S10: Over-represented Gene Ontology (GO) categories for co-expressed genes with similar direction of sex-bias in brain and tail. (XLSX 14 KB)

Additional file 13: Table S11: Guppy pigmentation orthologs and their positions on the female draft genome. Genes from the guppy reference transcriptome (GRT) encoding putative orthologous proteins to known candidates in pigment synthesis and pigment pattern formation in other vertebrates. The alignment percentage, sequence identity, and chromosomal positions of CDS from the GRT against the female draft genome are shown. (XLSX 23 KB)

Additional file 14: Table S12: Chromosomal distribution of sex-biased genes in brain, tail, and gonad. The total number of sex-biased transcripts per chromosome with their observed proportions and significance values for difference from expected proportions is described for genes with 1.2-fold (FDR < 0.05, < 0.1) and median-fold (FDR < 0.1) difference in expression. (XLSX 33 KB)

## Abbreviations

cDNA: Complementary DNA
CDS: Coding sequences
EST: Expressed sequence tags
QTL: Quantitative trait loci
SNP: Single nucleotide polymorphism
ORF: Open reading frame
F_adult_: Female adult
M_adult_: Male adult
F_embryo_: Female embryo
M_embryo_: Male embryo
F_brain_: Female brain
M_brain_: Male brain
F_tail_: Female tail
M_tail_: Male tail
F_gonad_: Female gonad
M_gonad_: Male gonad
GRT: Guppy reference transcriptome
bp: Base pair
GO: Gene ontology
FC: Fold change
CPM: Counts per million
FDR: False discovery rate
FPKM: Fragments per kilo base per million
*d*_*S*_: Number of synonymous substitutions per synonymous site
*d*_*N*_: Number of non-synonymous substitutions per non-synonymous site
UTR: Untranslated region
SDL: Sex determining locus
ECM: Extracellular matrix
LG: Linkage group
No.: Number
NR: NCBI non-redundant protein database.

## References

[1] Rowe L, Day T (2006). Detecting sexual conflict and sexually antagonistic coevolution. Philos Trans R Soc Lond Ser B Biol Sci.

[2] Lande R (1980). Sexual dimorphism, sexual selection, and adaptation in polygenic characters. Evolution; Int J of organic evolution.

[3] Hedrick AV, Temeles EJ (1989). The evolution of sexual dimorphism in animals: hypotheses and tests. Trends Ecol Evol.

[4] Rice WR (1984). Sex chromosomes and the evolution of sexual dimorphism. Evolution; Int J Organic Evolution.

[5] Rhen T (2000). Sex-limited mutations and the evolution of sexual dimorphism. Evolution; Int J Organic Evolution.

[6] Mank JE (2009). Sex chromosomes and the evolution of sexual dimorphism: lessons from the genome. Am Nat.

[7] Connallon T, Clark AG (2010). Sex linkage, sex-specific selection, and the role of recombination in the evolution of sexually dimorphic gene expression. Evolution; Int J Organic Evolution.

[8] Ellegren H, Parsch J (2007). The evolution of sex-biased genes and sex-biased gene expression. Nat Rev Genet.

[9] Yang X, Schadt EE, Wang S, Wang H, Arnold AP, Ingram-Drake L, Drake TA, Lusis AJ (2006). Tissue-specific expression and regulation of sexually dimorphic genes in mice. Genome Res.

[10] Small CM, Carney GE, Mo Q, Vannucci M, Jones AG (2009). A microarray analysis of sex- and gonad-biased gene expression in the zebrafish: evidence for masculinization of the transcriptome. BMC Genomics.

[11] Mank JE, Hultin-Rosenberg L, Webster MT, Ellegren H (2008). The unique genomic properties of sex-biased genes: insights from avian microarray data. BMC Genomics.

[12] Pointer MA, Harrison PW, Wright AE, Mank JE (2013). Masculinization of gene expression is associated with exaggeration of male sexual dimorphism. PLoS Genet.

[13] Parisi M, Nuttall R, Edwards P, Minor J, Naiman D, Lu J, Doctolero M, Vainer M, Chan C, Malley J, Eastman S, Oliver B (2004). A survey of ovary-, testis-, and soma-biased gene expression in Drosophila melanogaster adults. Genome Biol.

[14] Xia Q, Cheng D, Duan J, Wang G, Cheng T, Zha X, Liu C, Zhao P, Dai F, Zhang Z (2007). Microarray-based gene expression profiles in multiple tissues of the domesticated silkworm, Bombyx mori. Genome Biol.

[15] Parsch J, Ellegren H (2013). The evolutionary causes and consequences of sex-biased gene expression. Nat Rev Genet.

[16] Haerty W, Jagadeeshan S, Kulathinal RJ, Wong A, Ravi Ram K, Sirot LK, Levesque L, Artieri CG, Wolfner MF, Civetta A, Singh RS (2007). Evolution in the fast lane: rapidly evolving sex-related genes in Drosophila. Genetics.

[17] Proschel M, Zhang Z, Parsch J (2006). Widespread adaptive evolution of Drosophila genes with sex-biased expression. Genetics.

[18] Meisel RP (2011). Towards a more nuanced understanding of the relationship between sex-biased gene expression and rates of protein-coding sequence evolution. Mol Biol Evol.

[19] Assis R, Zhou Q, Bachtrog D (2012). Sex-biased transcriptome evolution in Drosophila. Genome Biol Evolution.

[20] Ellegren H (2011). Emergence of male-biased genes on the chicken Z-chromosome: sex-chromosome contrasts between male and female heterogametic systems. Genome Res.

[21] Meisel RP, Malone JH, Clark AG (2012). Disentangling the relationship between sex-biased gene expression and X-linkage. Genome Res.

[22] Leder EH, Cano JM, Leinonen T, O'Hara RB, Nikinmaa M, Primmer CR, Merila J (2010). Female-biased expression on the X chromosome as a key step in sex chromosome evolution in threespine sticklebacks. Mol Biol Evol.

[23] Vicoso B, Kaiser VB, Bachtrog D (2013). Sex-biased gene expression at homomorphic sex chromosomes in emus and its implication for sex chromosome evolution. Proc Natl Acad Sci U S A.

[24] Whittle CA, Johannesson H (2013). Evolutionary dynamics of Sex-biased genes in a hermaphrodite fungus. Mol Biol Evol.

[25] Devlin RH, Nagahama Y (2002). Sex determination and sex differentiation in fish: an overview of genetic, physiological, and environmental influences. Aquaculture.

[26] Volff JN, Schartl M (2001). Variability of genetic sex determination in poeciliid fishes. Genetica.

[27] Schultheis C, Böhne A, Schartl M, Volff JN, Galiana-Arnoux D (2009). Sex determination diversity and Sex chromosome evolution in poeciliid fish. Sexual Dev.

[28] Evans JP, Pilastro A (2011). Ecology and Evolution of Poeciliid Fishes.

[29] Endler J (1983). Natural and sexual selection on color patterns in poeciliid fishes. Environ Biol Fish.

[30] Bisazza A, Pilastro A (1997). Small male mating advantage and reversed size dimorphism in poeciliid fishes. J Fish Biol.

[31] Winge Ö (1927). The location of eighteen genes in Lebistes reticulatus. J of Gen.

[32] Reznick D, Endler JA (1982). The impact of predation on life-history evolution in trinidadian guppies (*Poecilia reticulata*). Evolution; Int J Organic Evolution.

[33] Fisher RAS (1931). The evolution of dominance. Biol Rev.

[34] Brooks R (2000). Negative genetic correlation between male sexual attractiveness and survival. Nature.

[35] Postma E, Spyrou N, Rollins LA, Brooks RC (2011). Sex-dependent selection differentially shapes genetic variation on and off the guppy Y chromosome. Evolution; Int J Organic Evolution.

[36] Houde AE, Endler JA (1990). Correlated evolution of female mating preferences and male color patterns in the guppy poecilia reticulata. Science.

[37] Brooks R, Endler JA (2001). Female guppies agree to differ: Phenotypic and genetic variation in mate-choice behavior and the consequences for sexual selection. Evolution; Int J Organic Evolution.

[38] Houde A (1997). Sex, Color, and Mate Choice in Guppies.

[39] Magurran AE (2001). Sexual conflict and evolution in Trinidadian guppies. Genetica.

[40] Magurran AE (1996). Battle of the sexes. Nature.

[41] Kemp DJ, Reznick DN, Grether GF, Endler JA (2009). Predicting the direction of ornament evolution in Trinidadian guppies (Poecilia reticulata). Proceedings Biol Sci / The Royal Soc.

[42] Griffiths SW, Magurran AE (1998). Sex and schooling behaviour in the Trinidadian guppy. Anim Behav.

[43] Brooks R, Endler JA (2001). Direct and indirect sexual selection and quantitative genetics of male traits in guppies (Poecilia reticulata). Evolution; Int J Organic Evolution.

[44] Reznick DNM,DB (1989). Review of Life-history Patterns in Poeciliid Fishes. In Ecology and Evolution of Livebearing Fishes (Poeciliidae).

[45] Tripathi N, Hoffmann M, Willing EM, Lanz C, Weigel D, Dreyer C (2009). Genetic linkage map of the guppy, Poecilia reticulata, and quantitative trait loci analysis of male size and colour variation. Proceedings Biol Sci/The Royal Soc.

[46] Dreyer C, Hoffmann M, Lanz C, Willing EM, Riester M, Warthmann N, Sprecher A, Tripathi N, Henz SR, Weigel D (2007). ESTs and EST-linked polymorphisms for genetic mapping and phylogenetic reconstruction in the guppy, Poecilia reticulata. BMC Genomics.

[47] Fraser BA, Weadick CJ, Janowitz I, Rodd FH, Hughes KA (2011). Sequencing and characterization of the guppy (Poecilia reticulata) transcriptome. BMC Genomics.

[48] Haskins CP, Haskins EF, McLaughlin JJA, Hewitt RE, Blair WF (1961). Polymorphism and population structure in Lebistes reticulatus, an ecological study. Vertebrate Speciation.

[49] Gordon SP, Lopez-Sepulcre A, Reznick DN (2012). Predation-associated differences in sex linkage of wild guppy coloration. Evolution; Int J Organic Evolution.

[50] Pruitt KD, Brown GR, Hiatt SM, Thibaud-Nissen F, Astashyn A, Ermolaeva O, Farrell CM, Hart J, Landrum MJ, McGarvey KM, Murphy MR, O'Leary NA, Pujar S, Rajput B, Rangwala SH, Riddick LD, Shkeda A, Sun H, Tamez P, Tully RE, Wallin C, Webb D, Weber J, Wu W, DiCuccio M, Kitts P, Maglott DR, Murphy TD, Ostell JM (2014). RefSeq: an update on mammalian reference sequences. Nucleic Acids Res.

[51] Schartl M, Walter RB, Shen Y, Garcia T, Catchen J, Amores A, Braasch I, Chalopin D, Volff JN, Lesch KP, Bisazza A, Minx P, Hillier L, Wilson RK, Fuerstenberg S, Boore J, Searle S, Postlethwait JH, Warren WC (2013). The genome of the platyfish, Xiphophorus maculatus, provides insights into evolutionary adaptation and several complex traits. Nat Genet.

[52] Braasch I, Brunet F, Volff JN, Schartl M (2009). Pigmentation pathway evolution after whole-genome duplication in fish. Genome Biol Evolution.

[53] Tripathi N, Hoffmann M, Weigel D, Dreyer C (2009). Linkage analysis reveals the independent origin of Poeciliid sex chromosomes and a case of atypical sex inheritance in the guppy (Poecilia reticulata). Genetics.

[54] Subramanian S, Kumar S (2004). Gene expression intensity shapes evolutionary rates of the proteins encoded by the vertebrate genome. Genetics.

[55] Vijay N, Poelstra JW, Kunstner A, Wolf JB (2013). Challenges and strategies in transcriptome assembly and differential gene expression quantification. A comprehensive in silico assessment of RNA-seq experiments. Mol Ecol.

[56] Lu B, Zeng Z, Shi T (2013). Comparative study of de novo assembly and genome-guided assembly strategies for transcriptome reconstruction based on RNA-Seq. Sci China Life Sci.

[57] Bradnam KR, Fass JN, Alexandrov A, Baranay P, Bechner M, Birol I, Boisvert S, Chapman JA, Chapuis G, Chikhi R, Chitsaz H, Chou WC, Corbeil J, Del Fabbro C, Docking TR, Durbin R, Earl D, Emrich S, Fedotov P, Fonseca NA Ganapathy G, Gibbs RA, Gnerre S, Godzaridis E, Goldstein S, Haimel M, Hall G, Haussler D, Hiatt JB, Ho IY (2013). Assemblathon 2: evaluating de novo methods of genome assembly in three vertebrate species. GigaSci.

[58] Magurran AE (2005). Evolutionary Ecology:The Trinidadian Guppy.

[59] Reznick D (1983). The structure of guppy life histories: the tradeoff between growth and reproduction. Ecology.

[60] Kaslin J, Ganz J, Brand M (2008). Proliferation, neurogenesis and regeneration in the non-mammalian vertebrate brain. Philos Trans R Soc Lond Ser B Biol Sci.

[61] Le Page Y, Diotel N, Vaillant C, Pellegrini E, Anglade I, Merot Y, Kah O (2010). Aromatase, brain sexualization and plasticity: the fish paradigm. Eur J Neuro Sci.

[62] Wlodarczyk J, Mukhina I, Kaczmarek L, Dityatev A (2011). Extracellular matrix molecules, their receptors, and secreted proteases in synaptic plasticity. Dev Neurobiol.

[63] Fujioka H, Dairyo Y, Yasunaga K, Emoto K (2012). Neural functions of matrix metalloproteinases: plasticity, neurogenesis, and disease. Biochem Res Int.

[64] Reader SM, Laland KN (2000). Diffusion of foraging innovations in the guppy. Anim Behav.

[65] Magurran AE, Garcia CM (2000). Sex differences in behaviour as an indirect consequence of mating system. J Fish Biol.

[66] Mensah E, Volkoff H, Unniappan S, Hökfelt T (2010). Galanin Systems in Non-mammalian Vertebrates with Special Focus on Fishes. Galanin.

[67] Cornbrooks EB, Parsons RL (1991). Sexually dimorphic distribution of a galanin-like peptide in the central nervous system of the teleost fish Poecilia latipinna. J Comp Neurol.

[68] Cornbrooks EB, Parsons RL (1991). Source of sexually dimorphic galanin-like immunoreactive projections in the teleost fish Poecilia latipinna. J Comp Neurol.

[69] Li MH, Yang HH, Li MR, Sun YL, Jiang XL, Xie QP, Wang TR, Shi HJ, Sun LN, Zhou LY, Wang DS (2013). Antagonistic roles of Dmrt1 and Foxl2 in Sex differentiation via estrogen production in tilapia as demonstrated by TALENs. Endocrinology.

[70] Ijiri S, Kaneko H, Kobayashi T, Wang DS, Sakai F, Paul-Prasanth B, Nakamura M, Nagahama Y (2008). Sexual dimorphic expression of genes in gonads during early differentiation of a teleost fish, the Nile tilapia Oreochromis niloticus. Biol Reprod.

[71] Sinclair A, Smith C (2009). Females battle to suppress their inner male. Cell.

[72] Uhlenhaut NH, Treier M (2011). Forkhead transcription factors in ovarian function. Reproduction.

[73] Thibault RE, Schultz RJ (1978). Reproductive adaptations among viviparous fishes (Cyprinodontiformes: Poeciliidae). Evolution; Int J Organic Evol.

[74] Turner CL (1940). Pseudoamnion, pseudochorion, and follicular pseudoplacenta in poeciliid fishes. J Morphol.

[75] Jollie WP, Jollie LG (1964). The fine structure of the ovarian follicle of the ovoviviparous poeciliid fish, Lebistes reticulatus. I. Maturation of follicular epithelium. J Morphol.

[76] Parisi M, Nuttall R, Naiman D, Bouffard G, Malley J, Andrews J, Eastman S, Oliver B (2003). Paucity of genes on the Drosophila X chromosome showing male-biased expression. Science.

[77] Khil PP, Smirnova NA, Romanienko PJ, Camerini-Otero RD (2004). The mouse X chromosome is enriched for sex-biased genes not subject to selection by meiotic sex chromosome inactivation. Nat Genet.

[78] Traut W, Winking H (2001). Meiotic chromosomes and stages of sex chromosome evolution in fish: zebrafish, platyfish and guppy. Chromosome Res.

[79] Mank JE, Hultin-Rosenberg L, Axelsson E, Ellegren H (2007). Rapid evolution of female-biased, but not male-biased, genes expressed in the avian brain. Mol Biol Evol.

[80] Grath S, Parsch J (2012). Rate of amino acid substitution is influenced by the degree and conservation of male-biased transcription over 50 myr of Drosophila evolution. Genome Biol Evolution.

[81] Mank JE, Ellegren H (2009). Are sex-biased genes more dispensable?. Biol Lett.

[82] Mank JE, Nam K, Brunstrom B, Ellegren H (2010). Ontogenetic complexity of sexual dimorphism and sex-specific selection. Mol Biol Evol.

[83] Swanson WJ, Vacquier VD (2002). The rapid evolution of reproductive proteins. Nat Rev Genet.

[84] Gavrilets S (2000). Rapid evolution of reproductive barriers driven by sexual conflict. Nature.

[85] Miller GT, Pitnick S (2002). Sperm-female coevolution in Drosophila. Science.

[86] Ludlow AM, Magurran AE (2006). Gametic isolation in guppies (Poecilia reticulata). Proceedings Biol Sci / The Royal Soc.

[87] Price AC, Helen Rodd F (2006). The effect of social environment on male–male competition in guppies (Poecilia reticulata). Ethology.

[88] Herdman EJE, Kelly CD, Godin J-GJ (2004). Male mate choice in the guppy (Poecilia reticulata): Do males prefer larger females as mates?. Ethology.

[89] Reznick DN, Shaw FH, Rodd FH, Shaw RG (1997). Evaluation of the rate of evolution in natural populations of guppies (Poecilia reticulata). Science.

[90] Mank JE, Hultin-Rosenberg L, Zwahlen M, Ellegren H (2008). Pleiotropic constraint hampers the resolution of sexual antagonism in vertebrate gene expression. Am Nat.

[91] Postlethwait J, Amores A, Cresko W, Singer A, Yan Y-L (2004). Subfunction partitioning, the teleost radiation and the annotation of the human genome. Trends Genet.

[92] Willing EM, Bentzen P, van Oosterhout C, Hoffmann M, Cable J, Breden F, Weigel D, Dreyer C (2010). Genome-wide single nucleotide polymorphisms reveal population history and adaptive divergence in wild guppies. Mol Ecol.

[93] Martyn U, Weigel D, Dreyer C (2006). In vitro culture of embryos of the guppy, Poecilia reticulata. Dev Dynam.

[94] Schneeberger K, Ossowski S, Lanz C, Juul T, Petersen AH, Nielsen KL, Jorgensen JE, Weigel D, Andersen SU (2009). SHOREmap: simultaneous mapping and mutation identification by deep sequencing. Nat Methods.

[95] Martin M (2011). Cutadapt removes adapter sequences from high-throughput sequencing reads. EMBnet.journal.

[96] Smeds L, Kunstner A (2011). ConDeTri–a content dependent read trimmer for Illumina data. PLoS One.

[97] Grabherr MG, Haas BJ, Yassour M, Levin JZ, Thompson DA, Amit I, Adiconis X, Fan L, Raychowdhury R, Zeng Q, Chen Z, Mauceli E, Hacohen N, Gnirke A, Rhind N, di Palma F, Birren BW, Nusbaum C, Lindblad-Toh K, Friedman N, Regev A (2011). Full-length transcriptome assembly from RNA-Seq data without a reference genome. Nat Biotechnol.

[98] Trapnell C, Williams BA, Pertea G, Mortazavi A, Kwan G, van Baren MJ, Salzberg SL, Wold BJ, Pachter L (2010). Transcript assembly and quantification by RNA-Seq reveals unannotated transcripts and isoform switching during cell differentiation. Nat Biotechnol.

[99] Trapnell C, Roberts A, Goff L, Pertea G, Kim D, Kelley DR, Pimentel H, Salzberg SL, Rinn JL, Pachter L (2012). Differential gene and transcript expression analysis of RNA-seq experiments with TopHat and Cufflinks. Nat Protocols.

[100] Li W, Godzik A (2006). Cd-hit: a fast program for clustering and comparing large sets of protein or nucleotide sequences. Bioinformatics.

[101] Fu L, Niu B, Zhu Z, Wu S, Li W (2012). CD-HIT: accelerated for clustering the next-generation sequencing data. Bioinformatics.

[102] Flicek P, Ahmed I, Amode MR, Barrell D, Beal K, Brent S, Carvalho-Silva D, Clapham P, Coates G, Fairley S, Fitzgerald S, Gil L, Garcia-Giron C, Gordon L, Hourlier T, Hunt S, Juettemann T, Kahari AK, Keenan S, Komorowska M, Kulesha E, Longden I, Maurel T, McLaren WM, Muffato M, Nag R, Overduin B, Pignatelli M, Pritchard B, Pritchard E (2013). Ensembl 2013. Nucleic Acids Res.

[103] Lechner M, Findeiss S, Steiner L, Marz M, Stadler PF, Prohaska SJ (2011). Proteinortho: detection of (co-)orthologs in large-scale analysis. BMC Bioinformatics.

[104] Altschul SF, Gish W, Miller W, Myers EW, Lipman DJ (1990). Basic local alignment search tool. J Mol Biol.

[105] Brown CT, Howe A, Zhang Q, Pyrkosz AB, Brom TH (2012). A Reference-Free Algorithm for Computational Normalization of Shotgun Sequencing Data.

[106] Langmead B, Salzberg SL (2012). Fast gapped-read alignment with Bowtie 2. Nat Methods.

[107] Gotz S, Garcia-Gomez JM, Terol J, Williams TD, Nagaraj SH, Nueda MJ, Robles M, Talon M, Dopazo J, Conesa A (2008). High-throughput functional annotation and data mining with the Blast2GO suite. Nucleic Acids Res.

[108] Wu TD, Watanabe CK (2005). GMAP: a genomic mapping and alignment program for mRNA and EST sequences. Bioinformatics.

[109] Roberts A, Pachter L (2013). Streaming fragment assignment for real-time analysis of sequencing experiments. Nat Methods.

[110] Gentleman RC, Carey VJ, Bates DM, Bolstad B, Dettling M, Dudoit S, Ellis B, Gautier L, Ge Y, Gentry J, Hornik K, Hothorn T, Huber W, Iacus S, Irizarry R, Leisch F, Li C, Maechler M, Rossini AJ, Sawitzki G, Smith C, Smyth G, Tierney L, Yang JY, Zhang J (2004). Bioconductor: open software development for computational biology and bioinformatics. Genome Biol.

[111] Robinson MD, McCarthy DJ, Smyth GK (2010). edgeR: a Bioconductor package for differential expression analysis of digital gene expression data. Bioinformatics.

[112] Robinson MD, Oshlack A (2010). A scaling normalization method for differential expression analysis of RNA-seq data. Genome Biol.

[113] Robinson MD, Smyth GK (2007). Moderated statistical tests for assessing differences in tag abundance. Bioinformatics.

[114] Alexa A, Rahnenfuhrer J (2010). TopGO: topGO: enrichment analysis for gene ontology. R package version 2.10.0.

[115] Benjamini Y, Hochberg Y (1995). Controlling the false discovery rate: a practical and powerful approach to multiple testing. J Roy Statist Soc Ser.

[116] R Core Team (2012). R: A Language and Environment for Statistical Computing. R Foundation for Statistical Computing.

[117] Katoh K, Standley DM (2013). MAFFT multiple sequence alignment software version 7: improvements in performance and usability. Mol Biol Evol.

[118] Yang Z (2007). PAML 4: phylogenetic analysis by maximum likelihood. Mol Biol Evol.

