# Supplementary material for: Transcriptome assemblies for studying sex-biased gene expression in the guppy, Poecilia reticulata
Source: BMC Genomics. 2014 May 26;15(1):400. doi: 10.1186/1471-2164-15-400 (PMC4059875; doi:10.1186/1471-2164-15-400)
Supplement: Supplementary file 1 — Additional file 1: Table S1: Description of Illumina cDNA libraries - sample preparation and sequenced datasets. (DOCX 27 KB) [file 12864_2014_6114_MOESM1_ESM.docx]

| **Table S1: Description of Illumina cDNA libraries - sample preparation and sequenced datasets** | | | | | | |
| --- | --- | --- | --- | --- | --- | --- |
| cDNA library (No. of individuals) | Organ(s) | Amount | Library preparation protocol | | No. of Read pairs after phred20 filtering | Dataset |
|  |  |  |  |  |  |  |
| Female adult: F_adult_ (9) | Brain (and Eyes), Liver (and spleen), skin, Tail, Ovaries | 15 μg of total RNA from each organ pooled (75µg total) prior to polyA+ purification | Paired End RNA library prepared with NEB RNA kit for Illumina (Each library sequenced separately on a single lane of Illumina GAII) | | 26,393,787 | Non-barcoded |
|  |  |  |  |  |  |  |
| Male Adult: M_adult_ (9) | Brain (and Eyes), Liver (and spleen), Skin, Tail, Testes |  |  |  | 29,481,947 |  |
| Female Embryo: F_embryo_(15) | Fin-clipped embryos | 75µg of total RNA used for polyA+ purification |  |  | 24,138,679 |  |
| Male Embryo: M_embryo_(15) | Fin-clipped embryos |  |  |  | 18,775,577 |  |
| Total read pairs : | | | | | 98,789,990 | |
| Female Brain: F_Brain_ (9,6**) | Brain and eyes | 3 µg of total RNA each | | Paired End RNA library prepared with Illumina TruSeq RNA kit : Each organ individually barcoded (3 x 13 libraries multiplexed and sequenced on 3 lanes of Illumina HiSeq) | 111,941,790, 79,016,273** | Barcoded* |
| Male Brain: M_Brain_ (6) | Brain and eyes |  |  |  | 70,950,871 |  |
| Female Tail: F_Tail_ (6) | Tail (skin, skeletal muscle, dorsal cord, bone and cartilage) | 2 µg of total RNA each | |  | 75,180,682 |  |
| Male Tail: M_Tail_ (6) | Tail (skin, skeletal muscle, dorsal cord, bone and cartilage) |  |  |  | 58,020,495 |  |
| Female Gonad: F_Gonad_ (6) | Ovaries | 1 µg of total RNA each | |  | 53,602,790 |  |
| Male Gonad: M_Gonad_ (6) | Testes |  |  |  | 53,065,339 |  |
| Total read pairs : | | | | | 422,761,967 | |
| ***Each barcoded library represents a single tissue from an individual guppy **All 9 female brain datasets (7 biological replicates and 2 technical replicates) were used for assembly but only 6 datasets from biological replicates were used for differential expression analysis** | | | | | | |
