# Supplementary material for: Transcriptome assemblies for studying sex-biased gene expression in the guppy, Poecilia reticulata
Source: BMC Genomics. 2014 May 26;15(1):400. doi: 10.1186/1471-2164-15-400 (PMC4059875; doi:10.1186/1471-2164-15-400)
Supplement: Supplementary file 4 — Additional file 4: Figure S1: Spearman’s correlation in expression. Hierarchical clustering of Spearman rank correlations. Coloring indicates spearman’s correlation in gene expression between samples from barcoded datasets (A) and all datasets (B). The dendrogram shows the agglomerative clustering (Ward’s) with the bootstrap values (percentage) showing the confidence in each branch. All samples show organ-specific clustering except for the gonads, that are most distinct from all other organs and cluster by sex. Female Brain FB; Male Brain MB; Female tail FT; Male tail MT; Female gonad FG; Male gonad MG; Female adult FAD; Male adult MAD; Female embryo FEM; Male embryo MEM. (PDF 471 KB) [file 12864_2014_6114_MOESM4_ESM.pdf]

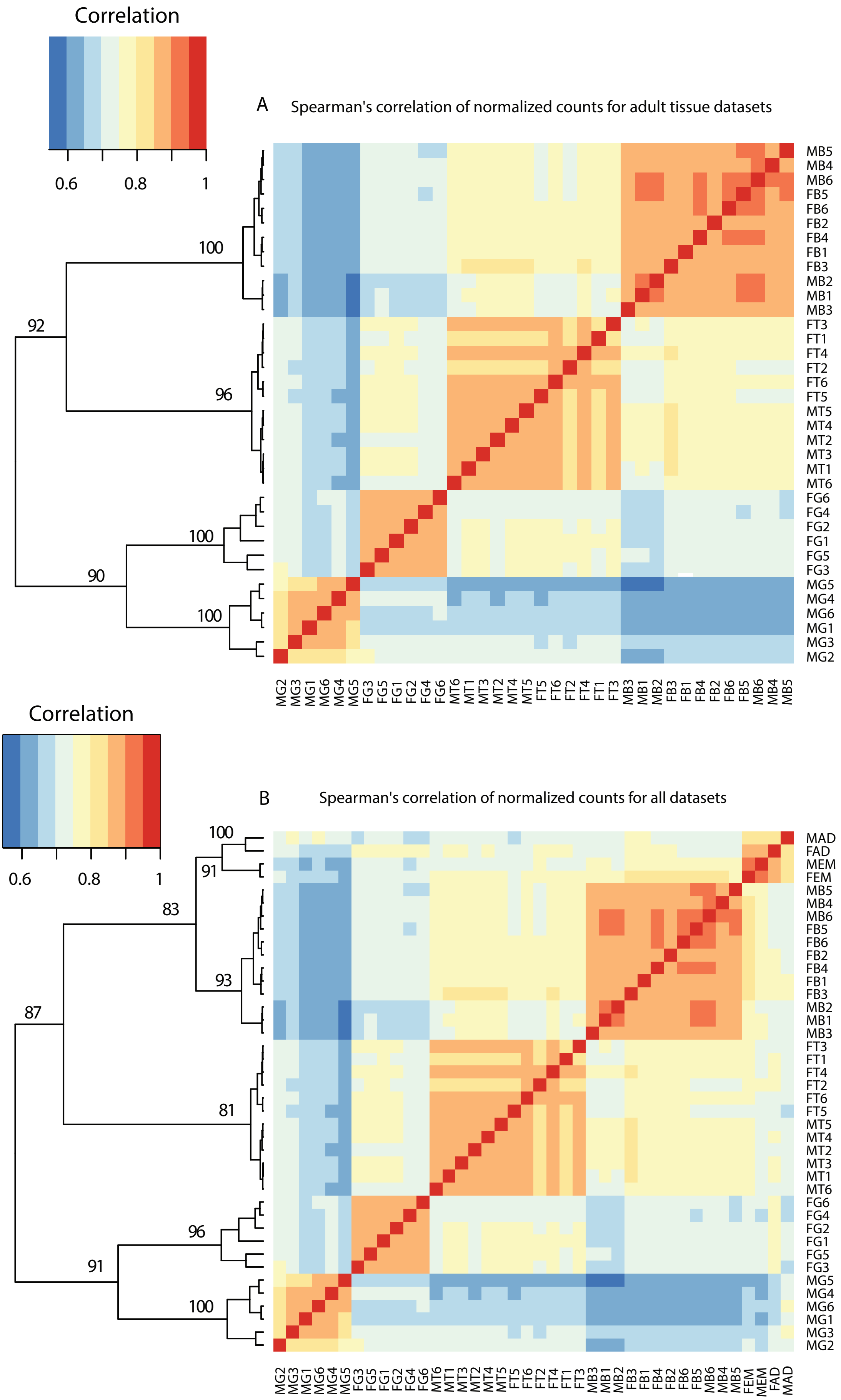

Figure S1: Hierarchical clustering of Spearman rank correlations. Colouring indicates spearman's correlation in gene expression between samples from barcoded datasets (A) and all datasets (B) . The dendrogram shows the agglomerative clustering (Ward's) with the bootstrap values (percentage) showing the confidence in each branch. All samples show organ-specific clustering except for the gonads, that are most distinct from all other organs and cluster by sex. Female Brain FB; Male Brain MB; Female tail FT; Male tail MT; Female gonad FG; Male gonad MG; Female adult FAD; Male adult MAD; Female embryo FEM; Male embryo MEM
