# Supplementary material for: Transcriptome assemblies for studying sex-biased gene expression in the guppy, Poecilia reticulata
Source: BMC Genomics. 2014 May 26;15(1):400. doi: 10.1186/1471-2164-15-400 (PMC4059875; doi:10.1186/1471-2164-15-400)
Supplement: Supplementary file 5 — Additional file 5: Figure S2: Expression differences between sexes. Expression differences between sexes. Distribution of expression statistics for genes with male-biased (MB), female-biased (FB), and unbiased (UB) expression in brain (grey), tail (yellow) and gonads (blue). (A) Boxplots show distribution of log2FC (Fold change: Male/Female). The lower median of each pair was used as cut-off for significant fold-change for that comparison (brain = 0.6; tail = 0.8; gonad = 1.8); (B) Boxplots show distribution of log2CPM (Counts per million) for sex-biased genes in each tissue pair. (C) Boxplots show distribution of coefficient of variation (CV) for all sex-biased genes (FDR <0.1) with greater than 1.2-fold change in expression and all unbiased genes (FDR > 0.1). (D) Boxplots show distribution of coefficient of variation (CV) for sex-biased genes (FDR <0.1) with greater than median-fold change in expression and all unbiased genes (FDR > 0.1). Outliers in Figure C and D are shown with black points. (PDF 3 MB) [file 12864_2014_6114_MOESM5_ESM.pdf]

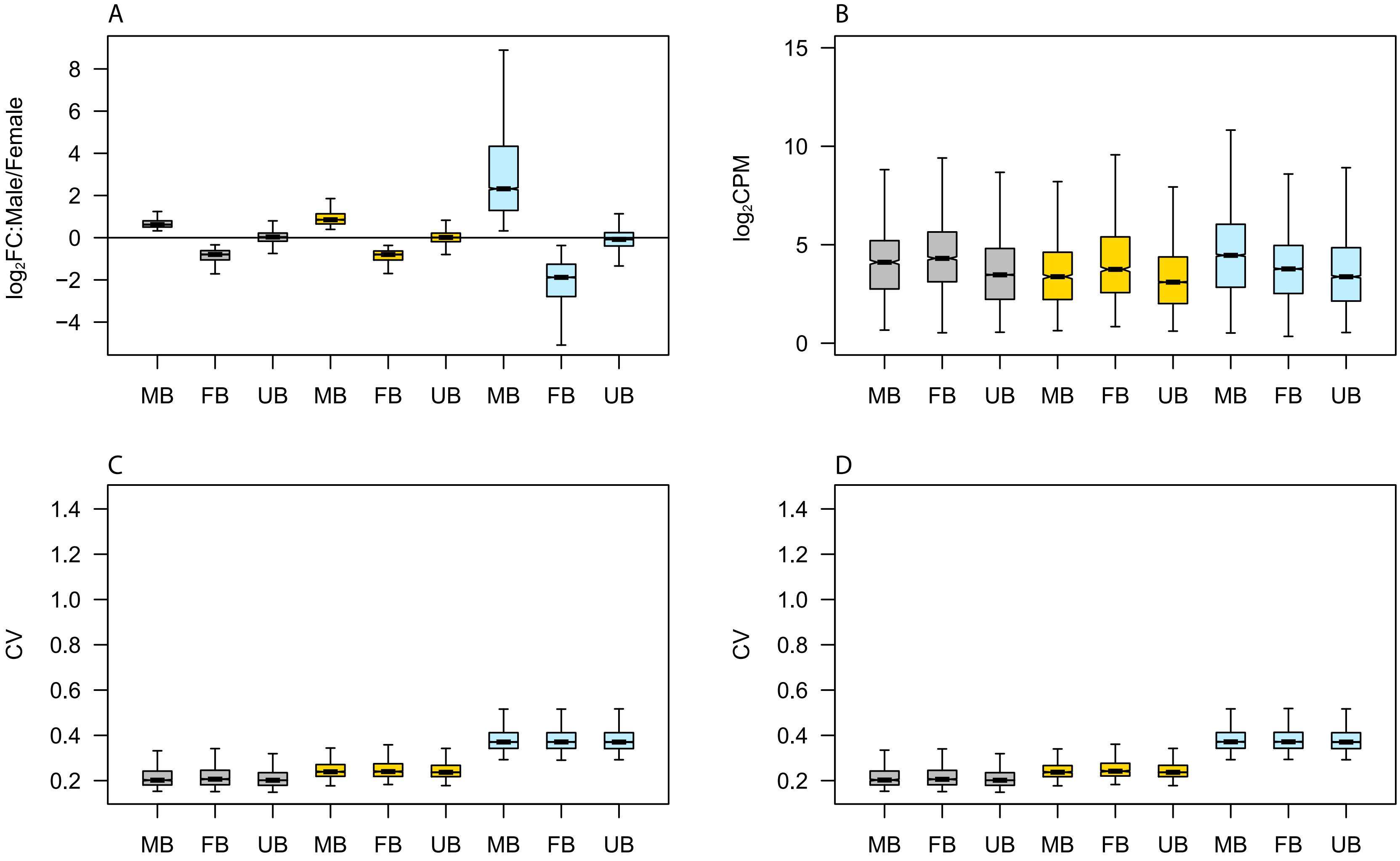

FigureS2: Expression differences between sexes. Distribution of expression statistics for genes with male-biased (MB), female-biased (FB), and unbiased (UB) expression in brain (grey), tail (yellow) and gonads (blue). (A) Boxplots show distribution of log_2FC (Fold change: Male/Female). The lower median of each pair was used as cut-off for significant fold-change for that comparison (brain = 0.6; tail = 0.8; gonad = 1.8); (B) Boxplots show distribution of log_2CPM (Counts per million) for sex-biased genes in each tissue pair.
(C) Boxplots show distribution of coefficient of variation (CV) for all sex-biased genes (FDR <0.1) with greater than 1.2-fold change in expression and all unbiased genes (FDR > 0.1). (D) Boxplots show distribution of coefficient of variation (CV) for sex-biased genes (FDR <0.1) with greater than median-fold change in expression and all unbiased genes (FDR > 0.1). Outliers in Figure C and D are shown with black points.
